# BMP-Non-Responsive Sca1^+^CD73^+^CD44^+^ Mouse Bone Marrow Derived Osteoprogenitor Cells Respond to Combination of VEGF and BMP-6 to Display Enhanced Osteoblastic Differentiation and Ectopic Bone Formation

**DOI:** 10.1371/journal.pone.0103060

**Published:** 2014-07-21

**Authors:** Vedavathi Madhu, Ching-Ju Li, Abhijit S. Dighe, Gary Balian, Quanjun Cui

**Affiliations:** Orthopaedic Research Laboratories, Department of Orthopaedic Surgery, University of Virginia, Charlottesville, Virginia, United States of America; Georgia Regents University, United States of America

## Abstract

Clinical trials on fracture repair have challenged the effectiveness of bone morphogenetic proteins (BMPs) but suggest that delivery of mesenchymal stem cells (MSCs) might be beneficial. It has also been reported that BMPs could not increase mineralization in several MSCs populations, which adds ambiguity to the use of BMPs. However, an exogenous supply of MSCs combined with vascular endothelial growth factor (VEGF) and BMPs is reported to synergistically enhance fracture repair in animal models. To elucidate the mechanism of this synergy, we investigated the osteoblastic differentiation of cloned mouse bone marrow derived MSCs (D1 cells) *in vitro* in response to human recombinant proteins of VEGF, BMPs (-2, -4, -6, -9) and the combination of VEGF with BMP-6 (most potent BMP). We further investigated ectopic bone formation induced by MSCs pre-conditioned with VEGF, BMP-6 or both. No significant increase in mineralization, phosphorylation of Smads 1/5/8 and expression of the ALP, COL1A1 and osterix genes was observed upon addition of VEGF or BMPs alone to the cells in culture. The lack of CD105, Alk1 and Alk6 expression in D1 cells correlated with poor response to BMPs indicating that a greater care in the selection of MSCs is necessary. Interestingly, the combination of VEGF and BMP-6 significantly increased the expression of ALP, COL1A1 and osterix genes and D1 cells pre-conditioned with VEGF and BMP-6 induced greater bone formation *in vivo* than the non-conditioned control cells or the cells pre-conditioned with either VEGF or BMP-6 alone. This enhanced bone formation by MSCs correlated with higher CADM1 expression and OPG/RANKL ratio in the implants. Thus, combined action of VEGF and BMP on MSCs enhances osteoblastic differentiation of MSCs and increases their bone forming ability, which cannot be achieved through use of BMPs alone. This strategy can be effectively used for bone repair.

## Introduction

Injuries to the postnatal skeleton are repaired through natural healing which is a complex, well-orchestrated process that recapitulates the pathway of embryonic development. It involves a variety of cell types and signaling molecules. Deficiencies in mesenchymal stem cells (MSCs) [1]–[2], angiogenesis induced by vascular endothelial growth factor (VEGF) [3]–[4] and bone morphogenetic proteins (BMPs) signaling [5]–[7] are associated with fractures that do not heal. It is estimated that of the 7.9 million fractures sustained each year in the United States, 5% to 20% result in delayed or impaired healing [8].

Clinical trials conducted using BMP-2 and BMP-7 to enhance bone repair showed that the method is not cost effective [9]–[11]. A recent review of 11 randomized controlled trials and 4 economical evaluations of BMPs for fracture repair concluded that only one study showed a difference in fracture healing between the BMP treated and control groups, but there was some suggestion that no second intervention was needed in the groups treated with BMP [10]. Several investigators have reported that BMPs fail to enhance mineralization and ALP expression in MSCs *in vitro* [12]–[21]; however the reasons that underlie the non-responsiveness of these cells are not understood. The limited success of clinical trials that used BMP-2 or BMP-7 could be due to suboptimal response of the osteoprogenitor cells as these stem cells play a pivotal role in BMP-induced bone repair [22]. The clinical trials using MSCs have shown promising results for fracture repair [23]–[24] which can be further enhanced through combined use of MSCs and BMPs if BMP-responsiveness of MSCs is better understood.

Combined delivery of MSCs, VEGF and BMPs (BMP-2, -4 and -7) has been immensely successful in enhancing fracture repair and bone formation in various animal models. We have systematically reviewed these studies recently [25]. These studies have shown that delivery of any single factor or combination of any two factors was less effective in inducing osteogenesis in comparison to that induced by MSCs, VEGF and BMP together. Synergistic enhancement of bone formation was BMP-type specific as VEGF and MSCs showed more influence on osteogenesis with BMP-4 than that with BMP-2 whereas there was no synergy with BMP-7 [26]–[28].

Since BMP-6 is reported to be more potent than BMP-2, -4 and -7 [29]–[30] and BMP-6 is resistant to inhibition by noggin while other BMPs are susceptible to noggin [31] which is predominantly expressed in non-union fractures [6]–[7], BMP-6 would be an ideal candidate to enhance fracture repair with VEGF and MSCs. We have demonstrated earlier that cloned mouse bone marrow derived mesenchymal stem cells (mBMMSCs) (D1 cells) co-expressing human VEGF and BMP-6 genes or VEGF and lim mineralization protein 1 (LMP1) genes induce significantly greater osteogenesis *in vivo* in comparison with that induced by D1 cells alone or by D1 cells expressing only one of those genes [32]–[33]. LMP-1 is a known downstream signal transducer of BMP-6 signaling pathway. To confirm these findings using primary cells, we transduced rat BMMSCs with adenoviral vector co-expressing VEGF and BMP-6 genes and showed that non-transduced rat BMMSCs failed to induce ectopic bone formation while transduced BMMSCs induced ectopic bone formation successfully [34]. We have also shown recently that simultaneous activation of intracellular VEGF and BMP-6 pathways enhances osteogenic differentiation of human adipose derived stem cells (hADSCs) [35]. However, the exact mechanism of enhanced bone formation by transiently transfected D1 cells expressing VEGF and BMP-6 [32] or VEGF and LMP-1 [33] was not completely understood. It remained elusive as to what role was played by exogenously added D1 cells and what was contribution of VEGF and BMP-6 secreted by the cells in enhancing bone formation. To gain more detailed insight into this paradigm, we sought to determine role of exogenously added MSCs in this study. We examined if cross-talk between VEGF and BMP-6 signaling pathways enhances osteogenic differentiation of D1 cells in* vitro* using human recombinant proteins of VEGF and BMP-6. We also characterized D1 cells for expression of MSCs-specific surface markers, expression of VEGF and BMP receptors and investigated bone formation elicited by D1 cells *in vivo* after they were pre-conditioned with VEGF and BMP-6 in this study.

## Methods

### Ethics statement

8–10 weeks old Balb/c mice (Taconic, NY, USA) were housed in the SPF Vivarium at the University of Virginia, which is fully accredited by the American Association for Accreditation of Laboratory Animal Care. This study was carried out in strict accordance with the recommendations in the Guide for the Care and Use of Laboratory Animals of the National Institutes of Health under Public Health Assurance number A3245-01. The protocol was approved by the University of Virginia Institutional Animal Care and Use Committee (protocol number 3701). All surgeries were performed under general anesthesia, and post-operative analgesia was given to all animals to minimize suffering.

### Cells, media, growth factors and culture conditions

We used D1 cells that were isolated in our laboratory from a BALB/c mouse bone marrow [36]. The D1 cells are also available from American Type Culture Collection (ATCC) under the product number CRL-12424. The D1 cells (passage 5) were grown in Dulbecco’s Modified Eagle’s Medium (Gibco BRL, Gaithersburg, MD., USA) containing 10% fetal bovine serum (Hyclone Laboratories, Logan, VT., USA), 50 µg/ml sodium ascorbate, 100 IU/ml penicillin G, and 100 µg/ml streptomycin in a humidified atmosphere of 5% carbon dioxide at 37°C. The medium was designated as basal medium (BM) and to induce osteogenesis, 10 mM β-glycerophosphate was added to BM to prepare osteogenic medium (OM). For all the experiments related to osteogenesis, the D1 cells were seeded at a density of 0.5×10^4^ cells/cm^2^ in a 24-well plate. Human recombinant proteins BMP-2, -4, -6; VEGF (Prospec, Israel) and BMP-9 (Assay Designs/Enzo Life Sciences, USA) were added to OM at the specified concentrations. Culture medium was replaced twice a week. The mineralization was determined using OM supplemented with 1, 10 and 100 ng of VEGF or individual BMPs and OM supplemented with a combination of 10 ng of VEGF and 10 ng of BMP-6. To determine mRNA expression of runx2 and osterix in presence of BMPs, the cells were grown in BM, OM and OM supplemented with 10 ng of BMP proteins. The Smads 1/5/8 phosphorylation was quantified using cells grown in BM, OM, OM containing 10 ng VEGF or 10 ng individual BMPs or combination of 10 ng VEGF and 10 ng BMP-6. The influence of cross-talk between VEGF and BMP-6 pathways on expression of osteogenic genes was investigated in cells grown in BM, OM and OM supplemented with 10 ng VEGF or BMP-6 or both. To investigate if pre-conditioning of the cells improves their bone formation ability, cells were grown in OM, OM supplemented with 10 ng VEGF or BMP-6 or both for 7 days and then implanted in sub-cutaneous tissues of Balb/c mice.

### The staining of cytoplasm and the nuclei

The D1 cells were seeded at a density of 1×10^3^ cells/cm^2^ in a 24-well plate and maintained in BM for 4 days. At day 5, the cells were stained using the methods described earlier [35].

### Alizarin red and Sudan IV staining

Mineralization was quantified using alizarin red staining as described earlier [35]. To determine if the D1 cells are capable of differentiation along an adipogenic lineage, the cells were cultured in BM containing 10^−7^ M dexamethasone for 14 days to induce adipogenesis. The differentiated cells were stained with Sudan IV, a stain for fat, counterstained with hematoxylin, and visualized by light microscopy (20X).

### RNA extraction, preparation of cDNA, conventional PCR and real time PCR

The methods used for RNA extraction, cDNA and quantification of expression of osteogenic markers using real time PCR were described earlier [35] and the primer sequences used are described in Table 1 RNA extraction from the harvested implant was performed using a TissueLyser as described earlier [37].

**Table 1:** List of primers used in real time PCR.

Name	Sequence
18S Forward	5′-CGGCGACGACCCATTCGAAC-3′
18 S Reverse	5′-GAATCGAACCCTGATTCCCCGTC-3′
Alkaline Phosphatase Forward	5′-ACGAGATGCCACCAGAGG-3′
Alkaline Phosphatase Reverse	5′-ACGAGATGCCACCAGAGG-3′
Runx2 Forward	5′-TTATCAAGGGAATAGAGGG-3′
Runx2 Reverse	5′-AGGACAGAGGGAAACAAC-3′
Osterix Forward	5′-ACCAGGTCCAGGCAACAC-3′
Osterix Reverse	5′-GCAAAGTCAGATGGGTAAGT-3′
Dlx5 Forward	5′-GATCCCTATGACAGGAGTGGGAC-3′
Dlx5 Reverse	5′-GGACTCGAGATCTAATAAAGCGTC-3′
COL1A1 Forward	5′-CGCCATCAAGGTCTACTGC-3′
COL1A1 Reverse	5′-GAATCCATCGGTCATGCTCT-3′
OPG Forward	5′-GCTGAGTGTTTTGGTGGACAGTT-3′
OPG Reverse	5′-GCTGGAAGGTTTGCTCTTGTG-3′
RANKL Forward	5′-TGCAGCATCGCTCTGTTCC-3′
RANKL Reverse	5′-CCCACAATGTGTTGCAGTTCC-3′
CADM1 Forward	5′-ATTCTGGGCCGCTATTTTG-3′
CADM1 Reverse	5′-TGTCCTCCTTCTGCATTGATT-3′

A measure of the mRNA for BMP-receptors (ActR-I/Alk2, BMPR-IA/Alk3, BMPR-IB/Alk6), TGF-receptors (Alk1, TβR-I/Alk5), VEGF receptors (membrane VEGFR1/mFlt-1, soluble VEGFR1/sFlt-1, VEGFR2/Flk1,) was determined using a cDNA template and gene specific primers by conventional PCR. The amplified DNA products from the PCR reactions were resolved in a 1% agarose gel and stained with ethidium bromide. The primers used are described in Table 2.

**Table 2:** List of primers used in PCR.

Name	Sequence
Alk1 Forward	5′-GTGTGGCGGTCAAGATTTTC-3′
Alk1 Reverse	5′-GGTTAGGGATGGTGGGTG-3′
Alk2 Forward	5′-CTGGACCAGAGGAACAAAGG-3′
Alk2 Reverse	5′-GGCGGGGTCTTACACGTCA-3′
Alk3 Forward	5′-TTATTCTGCTGCTTGTGGTCTGTG-3′
Alk3 Reverse	5′-CTTTACATCCTGGGATTCAAC-3′
Alk5 Forward	5′-GCGAAGGCATTACAGTGTTCT-3′
Alk5 Reverse	5′-TCTGAAATGAAAGGGCGATCTAGTGATGG-3′
Alk6 Forward	5′-GAAGATCAAGTGAATGCTGCACAG-3′
Alk6 Reverse	5′-GAACCAGCTGGCTTCCTC-3′
VEGFR2 Forward	5′-AGAACACCAAAAGAGAGAGGAACG-3′
VEGFR2 Reverse	5′-GCACACAGGCAGAAACCAGTAG-3′
mFlt1 Forward	5′-GTCACAGATGTGCCGAATGG-3′
mFlt1 Reverse	5′-TGAGCGTGATCAGCTCCAGG-3′
sFlt1 Forward	5′-GTCACAGATGTGCCGAATGG-3′
sFlt1 Reverse	5′-TGACTTTGTGTGGTACAATC-3′
β-actin Forward	5′-GCTGTATTCCCCTCCATCCTG-3′
β-actin Reverse	5′- CACGGTTGGCCTTAGGGTTCAG-3′

### Fluorescence Activated Cell Sorting

The D1 cells were grown in BM and harvested after 5 days. A homogeneous suspension of cells in PBS was prepared, washed and then incubated with anti-mouse CD16/CD32 monoclonal antibody Clone 2.4G2 (BD Biosciences, MD, USA) to block the Fc receptors. The D1 cells (1×10^6^ cells) were stained using specific monoclonal antibodies for 30 minutes. The list of antibodies is described in Table 3. The cells were washed three times with a solution of 1% BSA in PBS and analyzed using a FACS caliber flow cytometer (BD Biosciences, MD, USA); the data was analyzed using FloJo software (TreeStar, OR, USA).

**Table 3:** List of antibodies used for fluorescence activated cell sorting.

Name	Clone	Label	Source
Anti Sca-1	D7	Phycoerythrin (PE)	eBiosciences
Anti CD105		Fluorescein (FITC)	R and D System
Anti CD90	30-H12	PE	eBiosciences
Anti CD73	TY/11.8	Alexa Fluor 647	Biolegend
Anti CD45	A20	FITC	eBiosciences
Anti CD34	RAM 34	FITC	eBiosciences
Anti CD44	IM7	PE	eBiosciences
Anti CD146	ME-9F1	PE	Biolegend
Anti CD133	13A4	Peridinin-chlorophyll-protein complex (PerCP)	eBiosciences
Anti Nestin	25	Alexa Fluor 647	BD Biosciences

### Western blotting

The D1 cells were grown in BM or OM or with OM supplemented with VEGF or one of the BMP proteins or a combination of VEGF and BMP-6 for 24 hours and then lysed with SDS sample buffer without bromophenol blue (125 mM Tris-HCl pH 6.8, 150 mM β-mercaptoethanol, 1% SDS and 20% glycerol) in the presence of 1X protease inhibitor cocktail (Santa Cruz Biotechnology) and 1 mM PMSF (Santa Cruz Biotechnology). The lysates were immediately placed on ice. The protein concentration was determined using a Bradford protein assay kit (Biorad) and equal amounts of total cell proteins were resolved on 8–12% SDS-polyacrylamide gels, electro-transferred to nitrocellulose membranes (Thermo Scientific), membranes were blocked with 5% non-fat dry milk in TBST (50 mM Tris, pH 7.6, 150 mM NaCl, 0.05% tween 20) for 1 hour at room temperature, washed and incubated overnight at 4°C in 5% BSA in TBST containing anti-phospho-Smad1/5/8 (1∶1000) or anti-GAPDH antibodies (1∶1000) (Cell Signaling, USA). The membranes were then incubated with HRP-conjugated secondary antibody (1∶2000 in 5% non-fat dry milk in TBST) (Cell Singling, USA) for 1 hour at room temperature followed by chemiluminescent substrate for HRP antibody (Thermo Scientific) and enhancer solution (Thermo Scientific) mixed in a 1∶1 ratio. The membranes were incubated in the dark with CL-Xposure films (Pierce) and the films were developed to visualize the bands. To measure the density of bands quantitatively, electronic images were generated by placing the X-ray films in a GS-800 calibrated densitometer (Biorad, USA); images were quantified using ImageJ software.

### Mice and osteogenesis induced by the D1 cells activated with VEGF or BMP-6 or the combination of VEGF and BMP-6

The D1 cells were grown in OM or OM supplemented with VEGF or BMP-6 or both for 1 week. After one week, D1 cells were harvested, suspended in PBS and were mixed (1∶1 volume ratio) with Matrigel (BD Biosciences, NJ, USA) at 4°C and kept chilled in a syringe until injected in the mice. The mice were anesthetized using intra-peritoneal injection of ketamine (80 mg/kg) and xylazine (10 mg/kg). D1 cells (passage 4, 1×10^6^ cells/0.3 mL) in Matrigel suspension were injected on the dorsum of experimental mice. The detailed procedure is described earlier [37]. The mice from each group were sacrificed at week 2 and week 4 and at each time point harvested implants (n = 8) were used for radiography and H and E staining.

### Radiographs

The details of the methods are described earlier [37]. At 2 and 4 weeks the implants were surgically removed from the subcutaneous tissue and placed on an x-ray film, radiographs were taken; the films were subsequently developed and placed into a GS-800 Calibrated Densitometer (Bio-rad, CA, USA) to convert the radiograph films into digital images. Density measurements of radiographs were performed with the ImageJ software.

### Histology & Microscopy

For histological analysis, implants retrieved at week 2 and week 4 were decalcified using 0.25 M EDTA and fixed in 10% neutral buffered formalin, then dehydrated and embedded in paraffin. Six micrometer sections were stained with hematoxylin and eosin and evaluated by light microscopy.

### Statistical analysis

All *in vitro* experiments were repeated three times and bone formation in the sub-cutaneous tissue was repeated two times. Data from one individual set are represented in the manuscript. Statistical analysis of the averages of optical densities measured at 405 nm for the Alizarin Red staining assay, average of densities from radiographs, band densities in the western blot analysis, and the relative gene expression from real time PCR was performed. To determine whether the differences between the means of different groups were statistically significant, one-way ANOVA was used, followed by the LSD test using SPSS 18.0 software. The OM group was treated as the control. Statistical significance level was set at p<0.05.

## Results

### *In vitro* mineralization of mesenchymal stem cells

**Figure 1:**
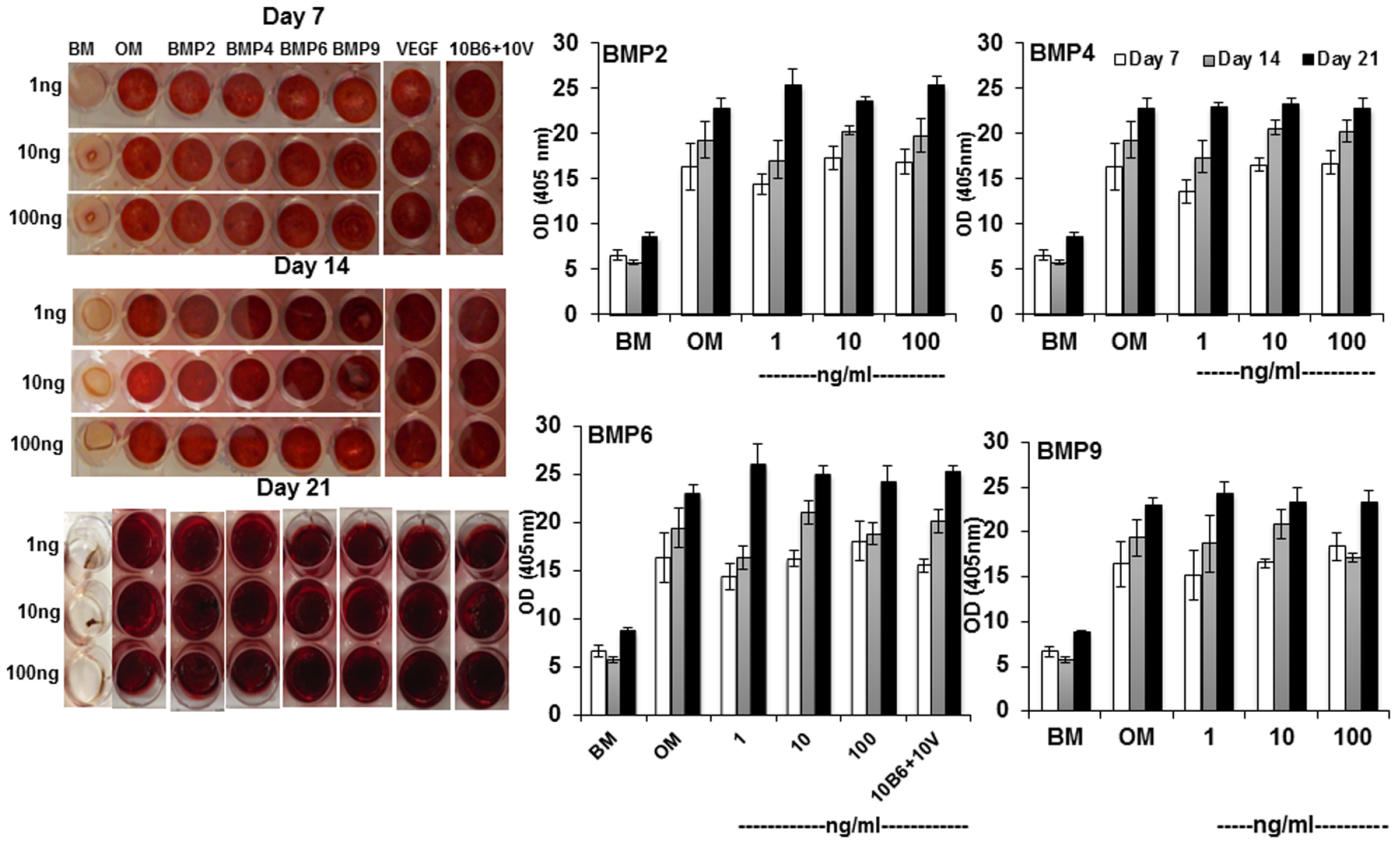
BMPs do not enhance mineralization. The mineralization of D1 cells was measured quantitatively using alizarin red staining. The cells were stained at day 7, 14 and 21 using alizarin red (left panel). The dye was extracted and intensity of color was quantified at 405 nm using a spectrophotometer (right panel).

Human recombinant proteins of VEGF or BMP-6 did not enhance mineralization of D1 cells at concentrations of 1 or 10 or 100 ng at day 7, 14 and 21 (Fig. 1). We therefore tested if other BMPs, BMP-2, -4 or -9 could enhance the mineralization. A similar trend was observed for all BMPs (Fig. 1). A combination of 10 ng VEGF and 10 ng BMP-6 also failed to enhance mineralization at day 7, 14 and 21.

### Expression of runx2 and osterix genes and activation of Smads 1/5/8

**Figure 2:**
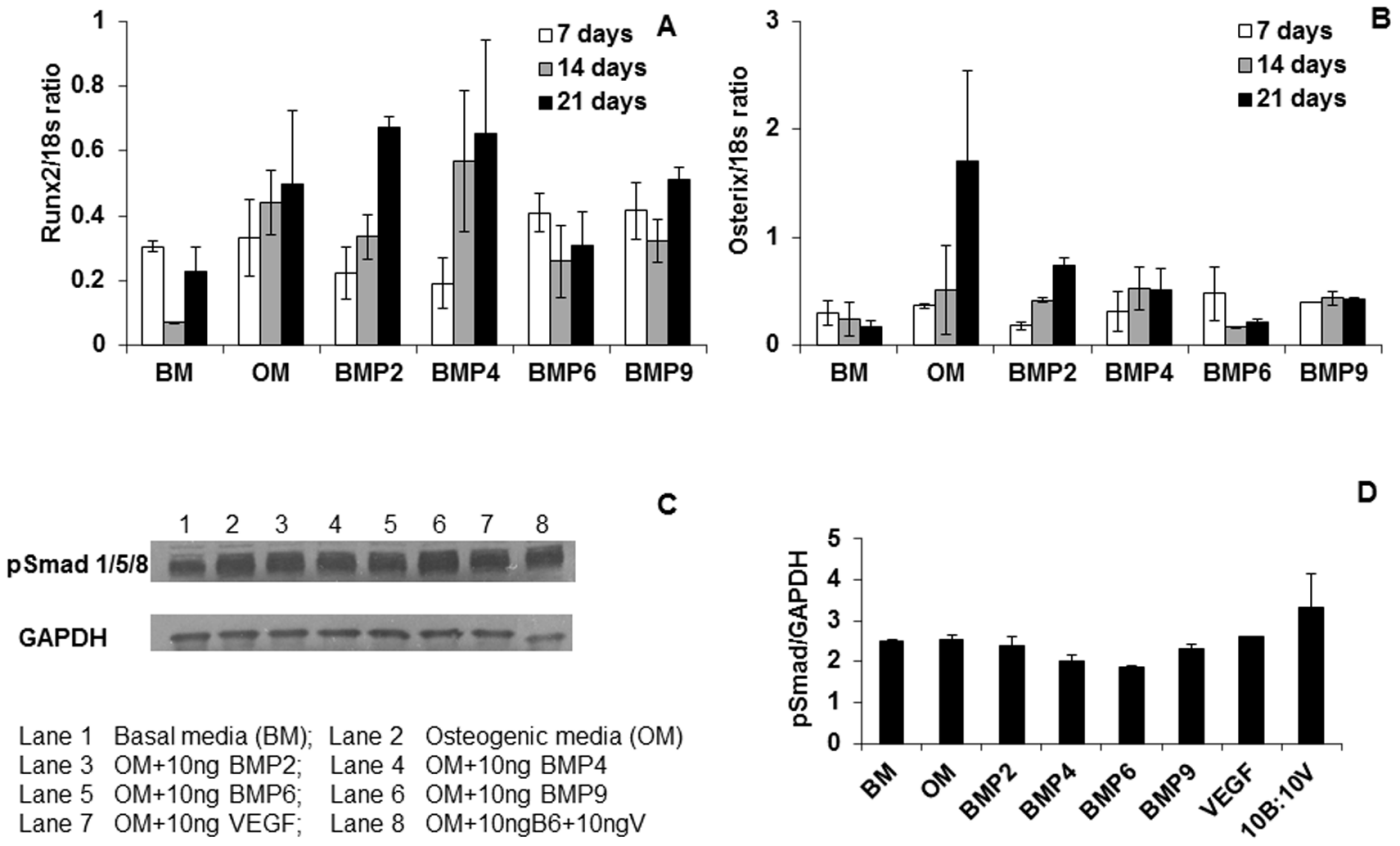
BMPs do not enhance Smad 1/5/8 phosphorylation and expression of runx2 and osterix genes. mRNA levels of runx2 (A) and osterix (B) were quantified using real time PCR. Smad phosphorylation was determined by western blots (C) and band intensity was quantified using ImageJ software (D).

Since VEGF, BMP-6 and other BMPs did not enhance mineralization, we determined if addition of these proteins would influence osteogenesis through modulation of the osteogenic transcription factors runx2 and osterix because both transcription factors are master regulators of osteogenesis. Surprisingly, transcription factor gene expression remained unchanged in response to the VEGF or BMP-6 or other BMPs (Fig. 2). Furthermore, Smads 1/5/8, which are downstream modulators of the BMP-signaling pathway, did not show increased phosphorylation upon treatment of D1 cells with VEGF or BMP-6 alone. Smad 1/5/8 phosphorylation increased when the cells were treated with the combination of VEGF and BMP-6 in comparison with all other groups. However, this increase was not statistically significant.

### Expression of mesenchymal stem cell surface markers and VEGF and BMP receptor genes

**Figure 3:**
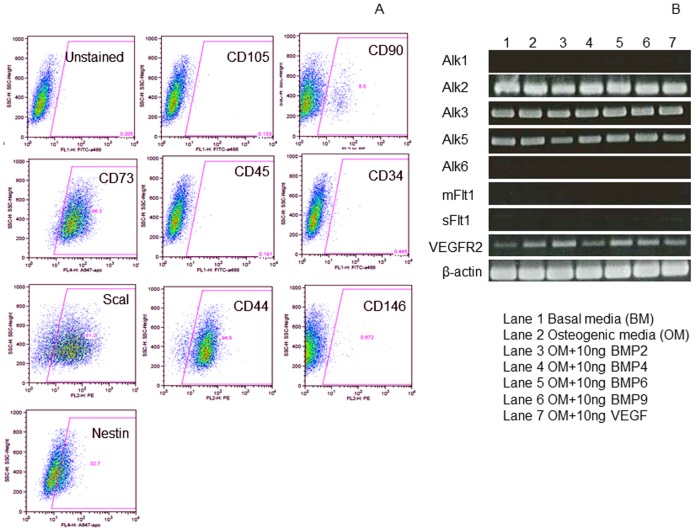
The D1 cells do not express CD105, Alk1 and Alk6 receptors. Using labeled monoclonal antibodies expression of stem cell markers (A) was determined by flow cytometry. Expression of VEGF and BMP receptors was determined by PCR followed by agarose gel electrophoresis (B).

To determine if lack of BMP-responsiveness of D1 cells correlates with expression of receptors, we determined expression of the receptor genes and found D1 cells expression of Alk2, Alk3 but a lack of expression of Alk1 and Alk6 (Fig. 3). It is known that BMP-2 and -4 bind to Alk3 as well as to Alk6; BMP-6 binds strongly to Alk2 and weakly to Alk6, and BMP-9 binds to Alk1 and Alk2. Therefore the D1 cells showed expression of at least one receptor for each of the BMPs that we tested i.e. BMP-2, -4, -6 and -9. In addition, D1 cells expressed the TGF-β binding receptor Alk5 (Fig. 3). The main receptor for VEGF165 is VEGFR2 (Flk-1) which was expressed in D1 cells but the cells did not express either the membrane form or the soluble form of VEGFR1 (Flt-1) (Fig. 3) that binds to VEGF thereby modulating the amount of VEGF available for VEGFR2. BMP-6 enhanced gene expression for VEGFR2 significantly. Cell surface analysis of the D1 cells showed a surface expression profile of CD105^−^CD90^−^CD73^+^CD45^−^CD34- (Fig. 3) which is not a classical CD105^+^CD90^+^CD73^+^CD45^−^CD34^−^ mesenchymal stem cell characteristic. However, the D1 cells expressed Sca-1 (∼80%), CD44 (∼80%) and nestin (∼30%) receptors that are known to be expressed by mesenchymal stem cells.

### D1 cells are capable of differentiation into adipogenic and osteogenic lineages in vitro

**Figure 4:**
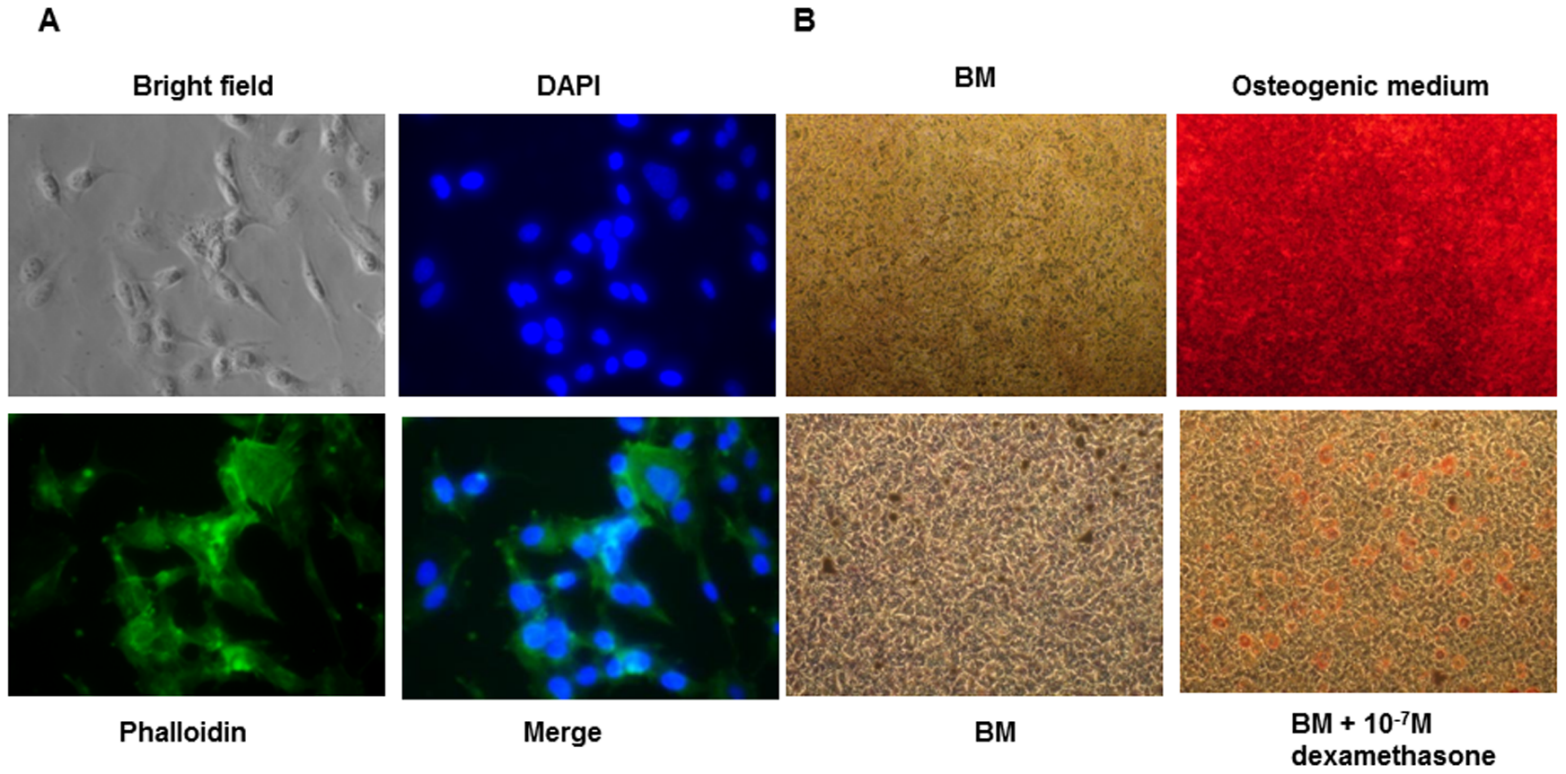
The D1 cells are multipotent. The D1 cells were stained with DAPI and phalloidin to visualize cell morphology (A). Osteogenesis was assayed by staining with Alizarin Red (B) and adipogenesis was assayed by staining with Sudan IV (C).

The D1 cells have large nuclei that occupy most of the cytoplasmic space (Fig. 4). In the presence of dexamethasone, D1 cells in culture differentiate into an adipogenic lineage and exhibit lipid vesicles. Similarly, in the presence of osteogenic medium, the D1 cells differentiate into the osteogenic lineage in vitro and display mineralization.

### Cross-talk between intracellular VEGF and BMP-6 pathways enhances expression of the ALP, COL1A1 and osterix genes

**Figure 5:**
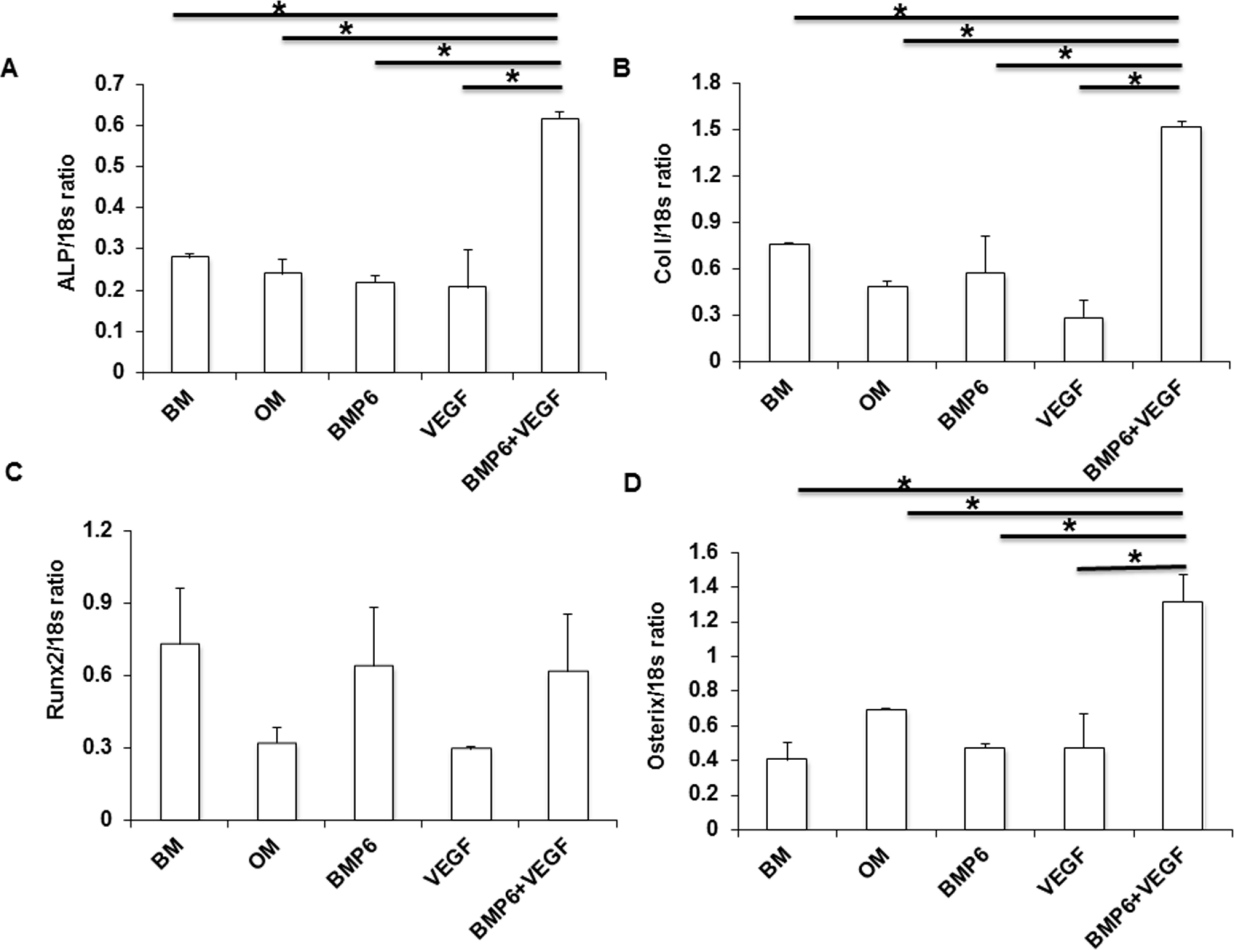
Combination of VEGF and BMP-6 enhances expression of osteogenic genes. Expression of ALP (A), COL1A1 (B), runx2 (C) and osterix (D) genes were determined by real time PCR at day 7. * denotes p<0.05.

Despite our observation that VEGF, BMP-6 and combination of VEGF plus BMP-6 did not enhance mineralization of D1 cells significantly, we considered that the combination of VEGF and BMP-6 might enhance expression of osteogenic markers ALP and COL1A1 genes. When the combination of 10 ng VEGF and 10 ng BMP-6 was added to the OM, ALP and COL1A1 gene expression at day 7 was increased significantly (Fig. 5) with a corresponding significant increase in osterix gene expression.

### Preconditioning of the D1 cells with the combination of VEGF and BMP-6 enhances osteogenesis *in vivo*

**Figure 6:**
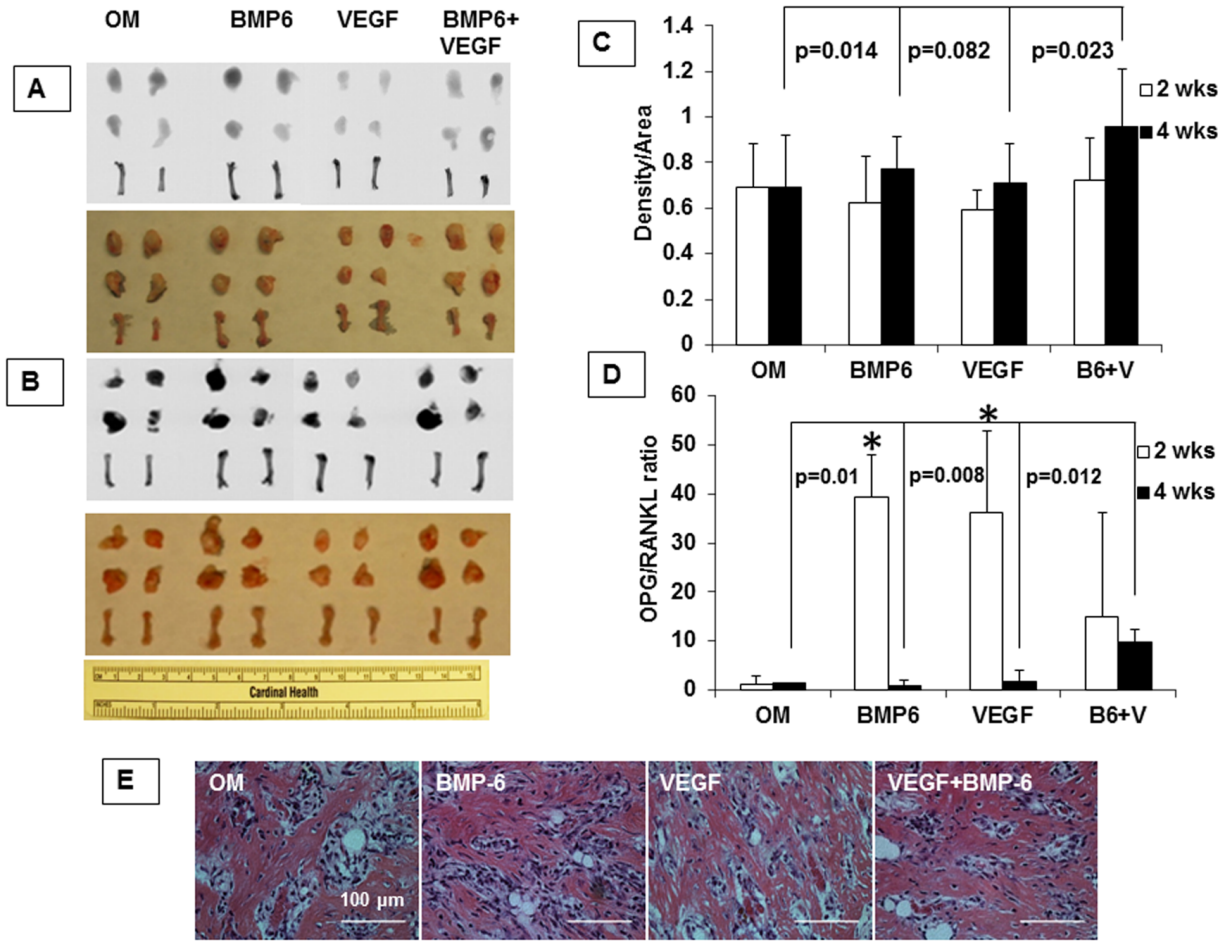
Preconditioning of BMP-non-responsive D1 cells with VEGF and BMP-6 enhances their ability to induce ectopic bone formation. The bone density of harvested implants was measured by radiography and Image J software (A-C). Representative images are shown in the figure. H and E staining revealed typical histology of bone (E). Real time PCR showed modulation of RANKL/OPG ratio (D). * denotes p<0.05 compared with OM group at weeks 2.

**Figure 7:**
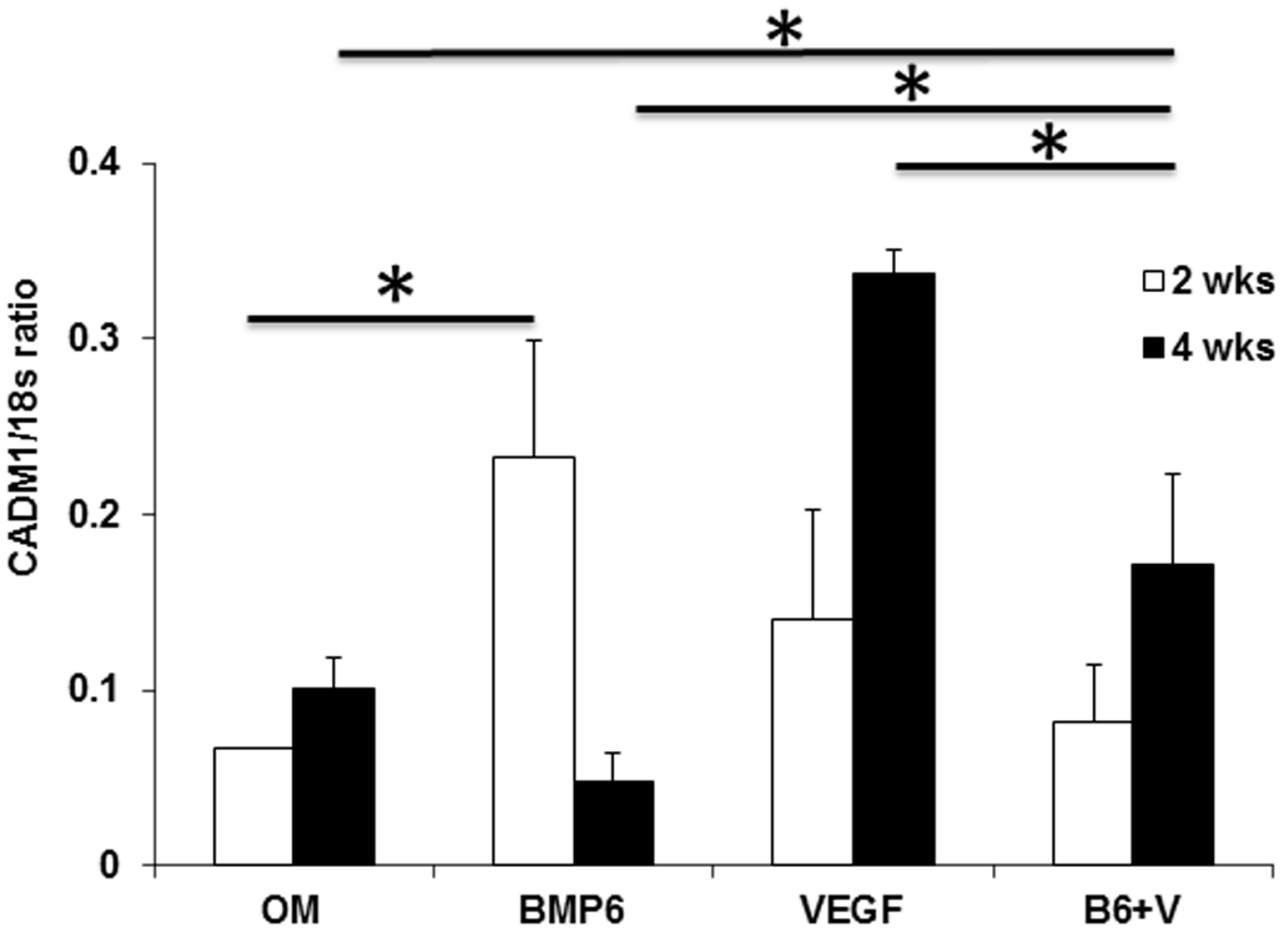
Enhanced expression of CADM1 in implants of D1 cells preconditioned with VEGF and BMP-6. RNA was isolated from the harvested implants and converted into cDNA. Using this cDNA as a template, mRNA expression of CADM1 was quantitatively determined in real time PCR. * denotes p<0.05.

**Figure 8:**
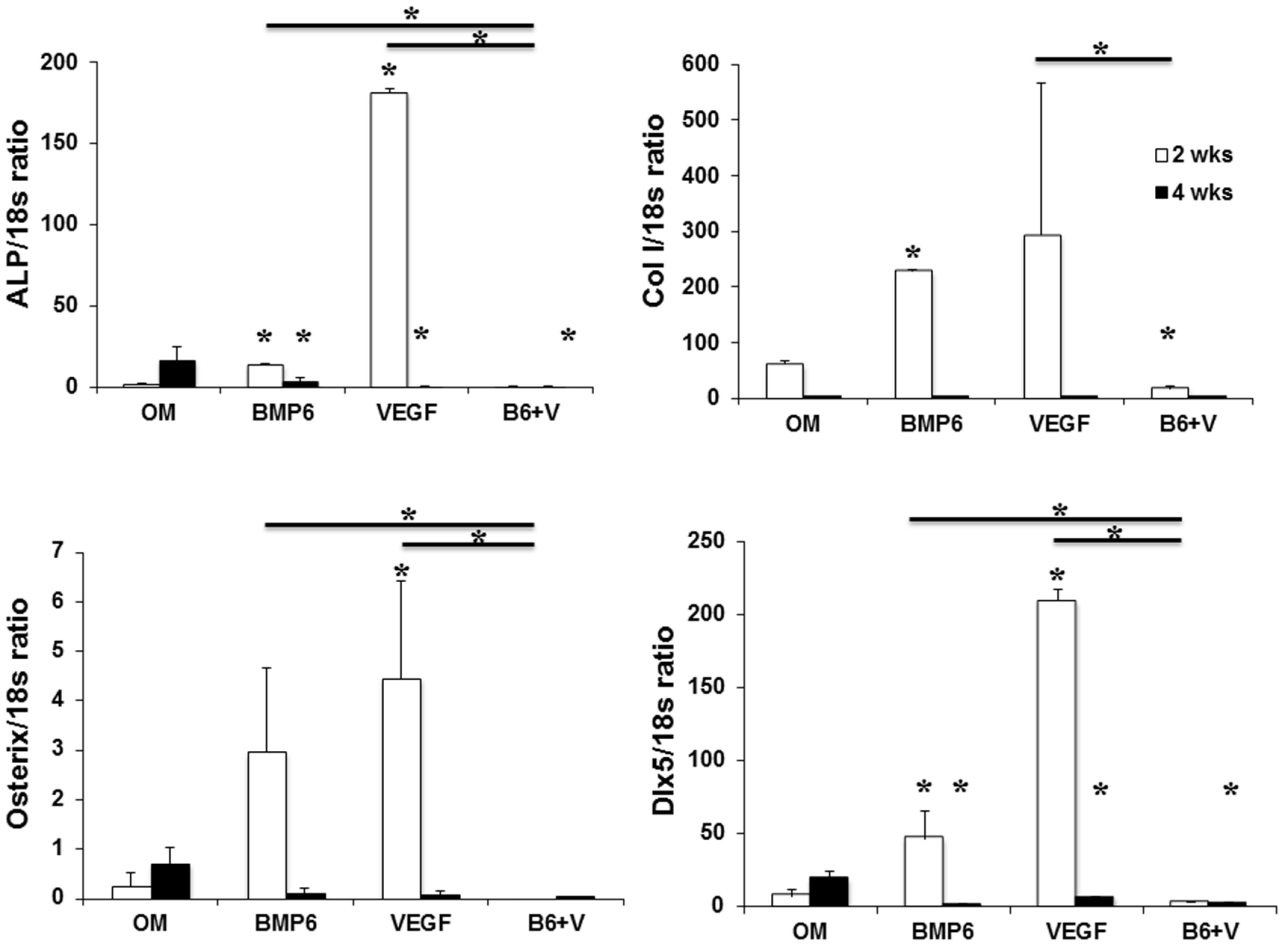
Expression of ALP and COL1 genes and genes for transcription factors osterix and Dlx5 in the implants of D1 cells. mRNA expression was quantified using gene specific primers and real time PCR. cDNA prepared from RNA isolated from the harvested implants was used as the template. * denotes p<0.05 compared with OM group of the respective time point, unless indicated otherwise by the horizontal lines.

Since the combination of 10 ng VEGF and 10 ng BMP-6 enhanced expressions of ALP, COL1A1 and osterix genes at week 1, we wanted to test if preconditioning of the D1 cells with the combination of VEGF and BMP-6 for 1 week could promote osteogenesis *in vivo*. The preconditioning with VEGF or BMP-6 or both did not enhance osteogenesis in comparison with OM group at week 2 (Fig. 6). At week 4, however, the D1 cells preconditioned with VEGF and BMP-6 induced significantly greater osteogenesis in comparison with that induced by other groups. The enhanced osteogenesis at week 4 correlated with higher ratio of mRNA levels of osteoprotegrin (OPG) to receptor activator of nuclear factor-kappaB ligand (RANKL) genes (Fig. 6) and higher expression of CADM1 (Fig. 7) in implants of D1 cells pre-conditioned with the combination of VEGF and BMP-6. We did not find any correlation between enhanced bone formation at week 4 and expression of ALP, COL1A1 genes or expression of genes of transcription factors osterix and Dlx5 in the implants at week 4 (Fig. 8). However, expression of ALP gene in the implants correlated with expression of osterix and Dlx5 genes while COL1A1 expression correlated with Dlx5 expression.

## Discussion

The purpose of this study was to gain insight into synergistic interaction between MSCs, VEGF and BMP in enhancing fracture repair. Various growth factors (BMPs, GDF-5, TGF-β, VEGF, angiopoetins, FGF, PDGF) and cell types (platelets, endothelial cells, macrophages, MSCs, chondrocytes, osteoclasts, osteoblasts) are known to be involved during fracture repair [25]. However, involvement of MSCs, VEGF and BMP is crucial as deficiencies in these factors can lead to fractures non-unions [1]–[7]. Interactions between these growth factors and cells during fracture repair are complex and are not completely understood. In the last decade several investigators have shown that exogenous addition MSCs, VEGF and BMP synergistically enhances bone formation but the exact mechanism remains elusive. It is difficult to explain this synergy through VEGF induced increased angiogenesis since our previous studies on synergistic aspect [32]–[33] revealed that increase in angiogenesis did not always correlate with increase in osteogenesis. While complete inhibition of angiogenesis is known to inhibit bone formation [3]–[4] the number of vessels and VEGF expression levels are reported to be similar in non-union fractures and fractures that heal [38]–[39]. Taken together, these facts suggest that synergistic enhancement of bone formation by MSCs, VEGF and BMP involves additional mechanisms than increase in angiogenesis which are not understood. Our data reveals these additional mechanisms as –1. Cross-talk between intracellular signaling pathways of VEGF and BMP-6 enhances osteoblastic differentiation of MSCs (Fig. 5) and 2. Paracrine factors produced by MSCs preconditioned with the combination of VEGF and BMP-6 modulate osteoclastogenesis through modulation of RANKL/OPG ratio (Fig. 6) and enhance osteogenic potential of MSCs as revealed by CADM1 expression (Fig. 7). CADM1 has been recently identified as a marker of higher bone forming capacity of MSCs [40]. The findings of this study shed light on possible mechanism for our earlier findings that D1 cells transiently (≤1 week) expressing VEGF and BMP-6 genes [32] or VEGF and LMP-1 genes [33] induced significantly greater bone formation at week 3 than that induced by D1 cells alone or D1 cells expressing any single gene. It appears from our present study (Fig. 6) and our previous findings [32]–[33] that transient VEGF and BMP-6 (or LMP-1) activation of D1 cells for about a week is sufficient to warrant superior bone formation. A recent study nicely demonstrated using conditional knock-out mice that intracellular levels of VEGF and VEGF signaling in MSCs control fate of differentiation of MSCs into osteogenic versus adipogenic lineage and this function of VEGF is quite distinct than that of secreted VEGF functions [41].

Synergistic enhancement of bone formation using MSCs, VEGF and BMP is reported to be cell-type specific as VEGF and BMP-4 co-expressing C2C12 cells made more bone in comparison with that made by NIH3T3 cells [42]. The D1 cells did not exhibit increased mineralization in response to BMPs (Fig. 1). This data is in agreement with reported findings that BMPs do not enhance mineralization of MSCs *in vitro* [12]–[21]. We also found that BMP-6 alone did not enhance expression of runx2, osterix, ALP, COL1A1 genes (Fig. 5) and phosphorylation of Smads 1/5/8 (Fig. 2) in D1 cells.

We investigated why D1 cells did not respond to BMPs. Although the D1 cells expressed the main receptors for BMPs, they did not express Alk1 and Alk6 (Fig. 3). Alk6 is not required for ALP expression and mineralization in MSCs [15], but we do not know if Alk6 is required for up regulation of runx2 and osterix genes and phosphorylation of Smads 1/5/8 in MSCs in response to BMP-6. Although the D1 cells were capable of differentiation into adipogenic and osteogenic lineages *in vitro* (Fig. 4) they did not express CD105 and CD90 receptors (Fig. 3) making it difficult to distinguish them as MSCs. Non-responsiveness of D1 cells to BMPs can be attributed to the lack of surface expression of CD105 because CD105 is the co-receptor for BMP-signaling [43].

Interestingly, we discovered that BMP-nonresponsive phenotype of D1 cells *in vitro* could be partially overcome with pre-conditioning using a combination of VEGF and BMP-6 (Fig. 5). Most importantly, ability of BMP-non-responsive D1 cells to induce bone formation *in vivo* was significantly enhanced if the cells were pre-conditioned with VEGF and BMP-6 through paracrine modulation of RANKL/OPG ratio (Fig. 6) and expression of CADM1 (Fig. 7). This strategy can be effectively used for enhancement of fracture repair.

## Conclusion

The data presented in this report demonstrates that: 1. osteoprogenitor cells that do not express CD105, Alk1 and Alk6 receptors do not respond well to BMPs *in vitro*, 2. These cells respond to a combination of VEGF and BMP-6 *in vitro,* moreover, VEGF signaling is required for a major response of MSCs to BMPs and, 3. Preconditioning of these cells with the combination of VEGF and BMP-6 enhances their ability to induce ectopic bone formation through paracrine modulation of the RANKL/OPG ratio and expression of CADM1. Overall, the data suggests that signaling interplay between the VEGF and the BMPs that are produced during fracture repair together with the endogenous or exogenously added MSCs is critical for bone repair. Therefore, greater care should be exercised while selecting MSCs for tissue engineering purposes since MSCs that do not express essential receptors might be less responsive to BMPs. Use of either MSCs alone, or VEGF or BMPs alone might not be suitable for bone repair but the combined use of MSCs, VEGF and BMP-6 is likely to be a useful approach for the development of therapies that would enhance fracture repair.
