# Correction: BMP-Non-Responsive Sca1^+^CD73^+^CD44^+^ Mouse Bone Marrow Derived Osteoprogenitor Cells Respond to Combination of VEGF and BMP-6 to Display Enhanced Osteoblastic Differentiation and Ectopic Bone Formation

**DOI:** 10.1371/journal.pone.0110204

**Published:** 2014-09-30

**Authors:** The PLOS ONE Staff

The label C is missing in Figure 4. The authors have provided a corrected version here.

**Figure 4:**
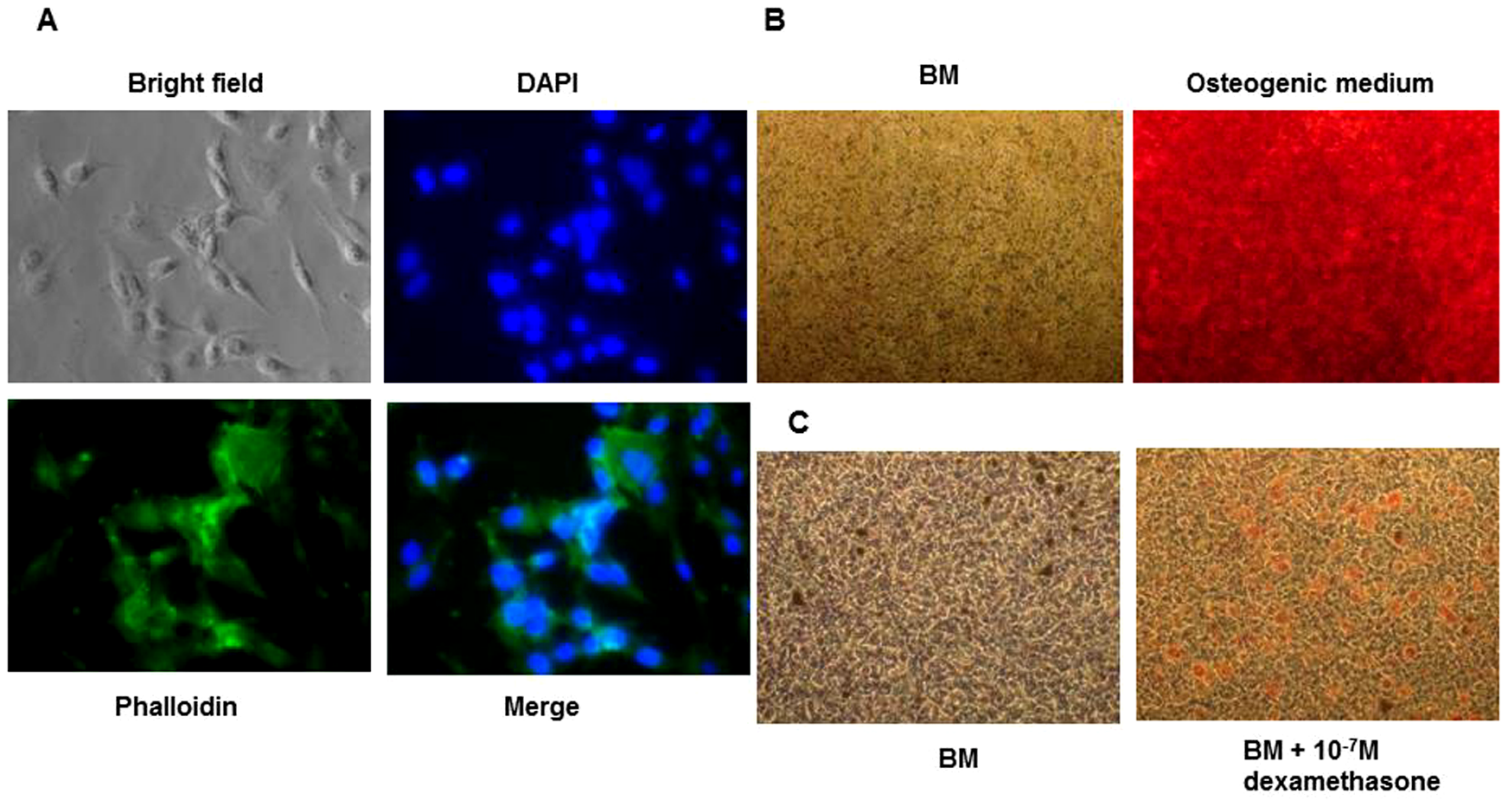
The D1 cells are multipotent. The D1 cells were stained with DAPI and phalloidin to visualize cell morphology (A). Osteogenesis was assayed by staining with Alizarin Red (B) and adipogenesis was assayed by staining with Sudan IV (C).
