# Correction: BMP-Non-Responsive Sca1^+^CD73^+^CD44^+^ Mouse Bone Marrow Derived Osteoprogenitor Cells Respond to Combination of VEGF and BMP-6 to Display Enhanced Osteoblastic Differentiation and Ectopic Bone Formation

**DOI:** 10.1371/journal.pone.0211782

**Published:** 2019-01-31

**Authors:** Vedavathi Madhu, Ching-Ju Li, Abhijit S. Dighe, Gary Balian, Quanjun Cui

After publication of this article [1], concerns were raised about Figs 1 and 2.

There were errors in the preparation of the left panel of Fig 1 which resulted in duplication of wells within the figure. A detailed visual guide to the errors generated while copying the wells from the original images is provided as Supporting Information (S1 File). Please see the corrected Fig 1 here, which was prepared by copying the first row of every experimental group for the sake of uniformity and clarity. The errors did not affect the data charts shown in Fig 1 nor the results and conclusions reported.

**Fig 1:**
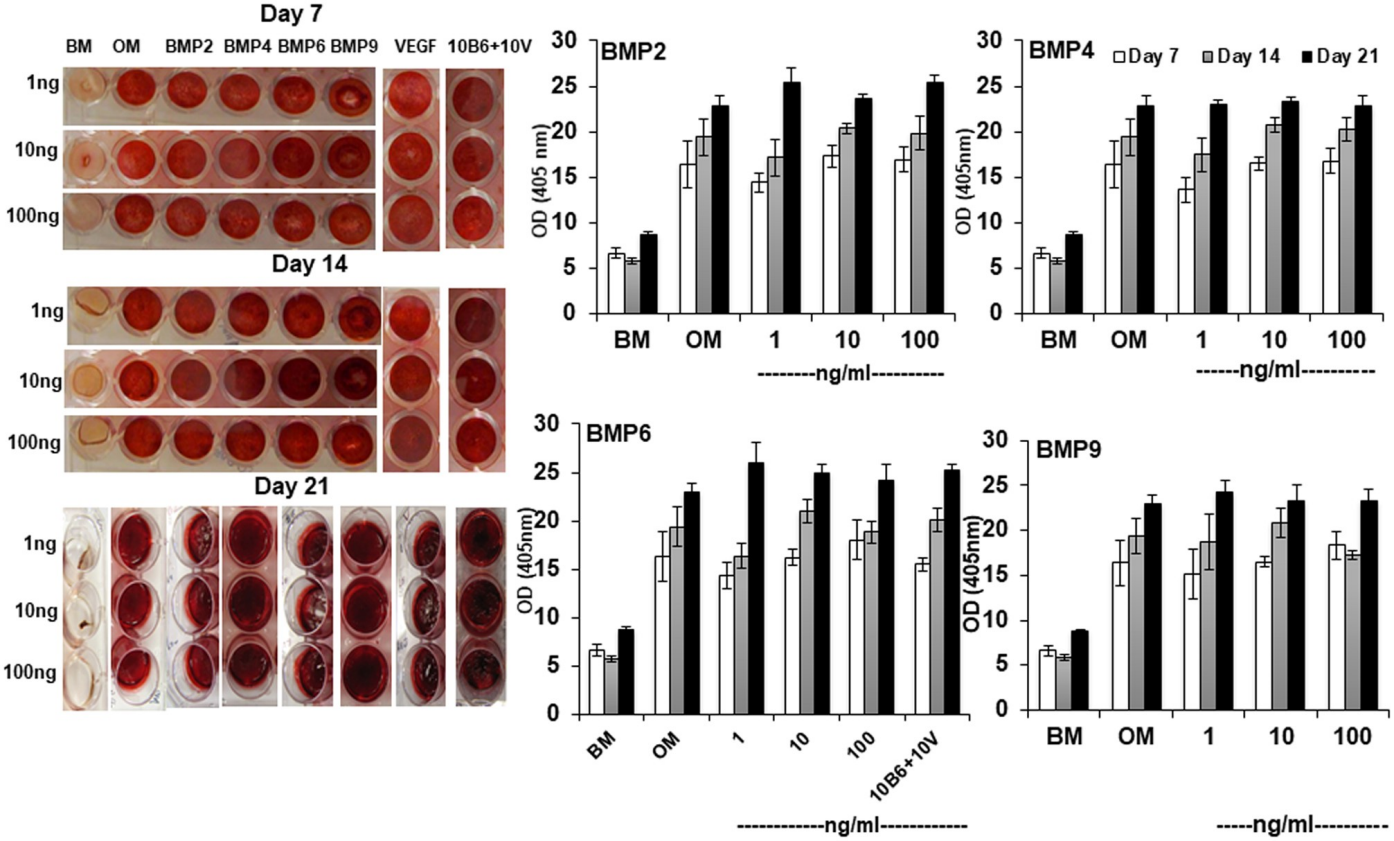
BMPs do not enhance mineralization. The mineralization of D1 cells was measured quantitatively using alizarin red staining. The cells were stained at day 7, 14 and 21 using alizarin red (left panel). The dye was extracted and intensity of color was quantified at 405 nm using a spectrophotometer (right panel).

In Fig 2, the splicing of lanes in the western blot (to remove three lanes from the original blot) was not clearly indicated. Please see the corrected Fig 2 and caption here, in which the splicing location is indicated by a vertical black line.

**Fig 2:**
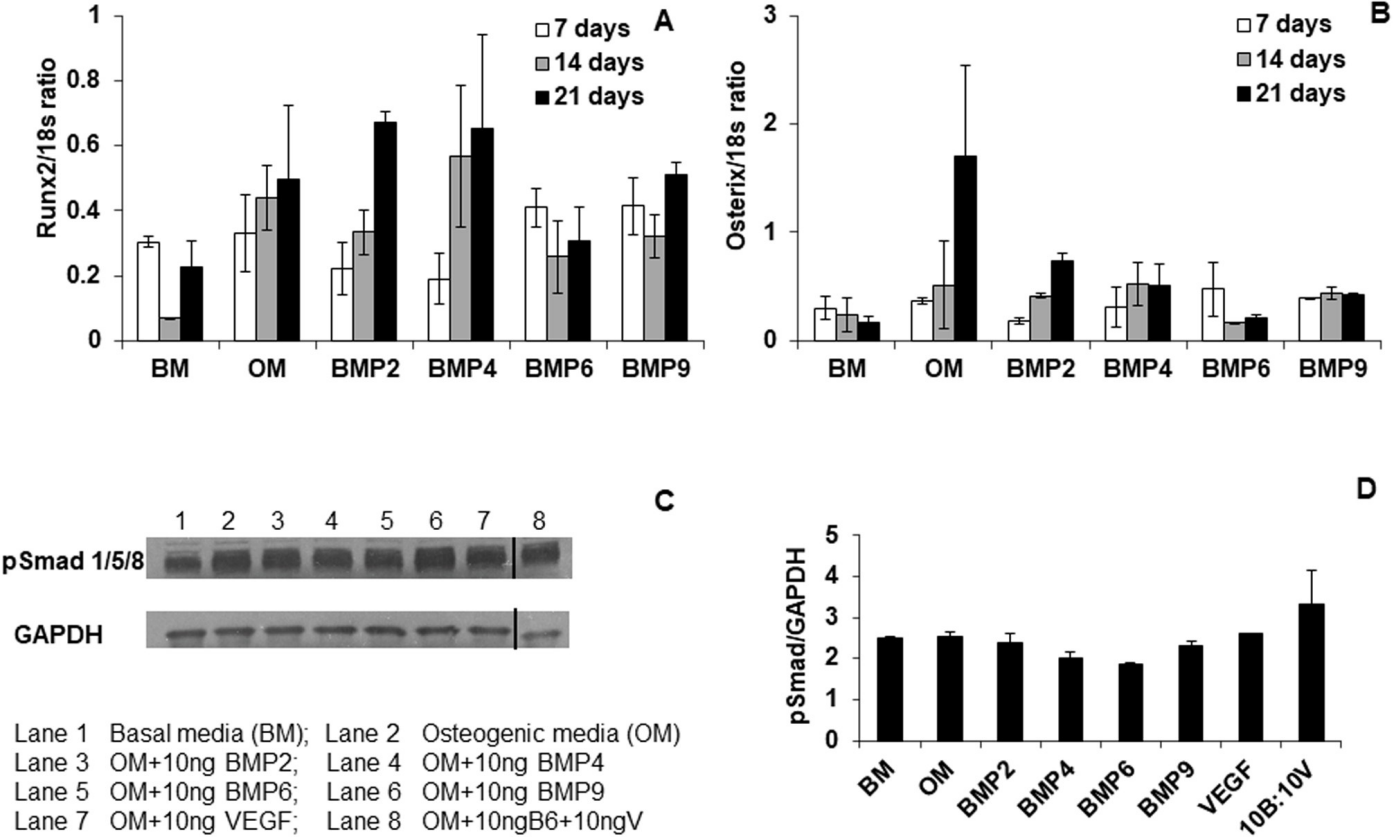
BMPs do not enhance Smad 1/5/8 phosphorylation and expression of runx2 and osterix genes. mRNA levels of runx2 (A) and osterix (B) were quantified using real time PCR. Smad phosphorylation was determined by western blots (C) and band intensity was quantified using ImageJ software (D). Three lanes were removed between lanes 7 and 8 to prepare the figure, this, is indicated by the vertical black line.

The original real time PCR data files and raw flow cytometry data are no longer available. The authors have provided the available original underlying data for Figs 1-8 as Supporting Information files (S1–S9 Data). This includes the uncropped images underlying Fig 1 and the uncropped Western blots underlying Fig 2.

## Supporting information

S1 File Guide to errors generated in Fig 1. (PDF)

S1 Data Data and figures underlying Fig 1. (ZIP)

S2 Data Data and figures underlying Fig 2. (ZIP)

S3 Data Figures underlying Fig 3. (PPTX)

S4 Data Figures underlying Fig 4. (PPTX)

S5 Data Data and figures underlying Fig 5. (ZIP)

S6 Data Data underlying Fig 6. (ZIP)

S7 Data Figures underlying Fig 6. (ZIP)

S8 Data Data underlying Fig 7. (ZIP)

S9 Data Data underlying Fig 8. (ZIP)
