# Supplementary material for: Correction: BMP-Non-Responsive Sca1+CD73+CD44+ Mouse Bone Marrow Derived Osteoprogenitor Cells Respond to Combination of VEGF and BMP-6 to Display Enhanced Osteoblastic Differentiation and Ectopic Bone Formation
Source: PLoS One. 2019 Jan 31;14(1):e0211782. doi: 10.1371/journal.pone.0211782 (PMC6355026; doi:10.1371/journal.pone.0211782)
Supplement: S1 File — (PDF) [file pone.0211782.s001.pdf]

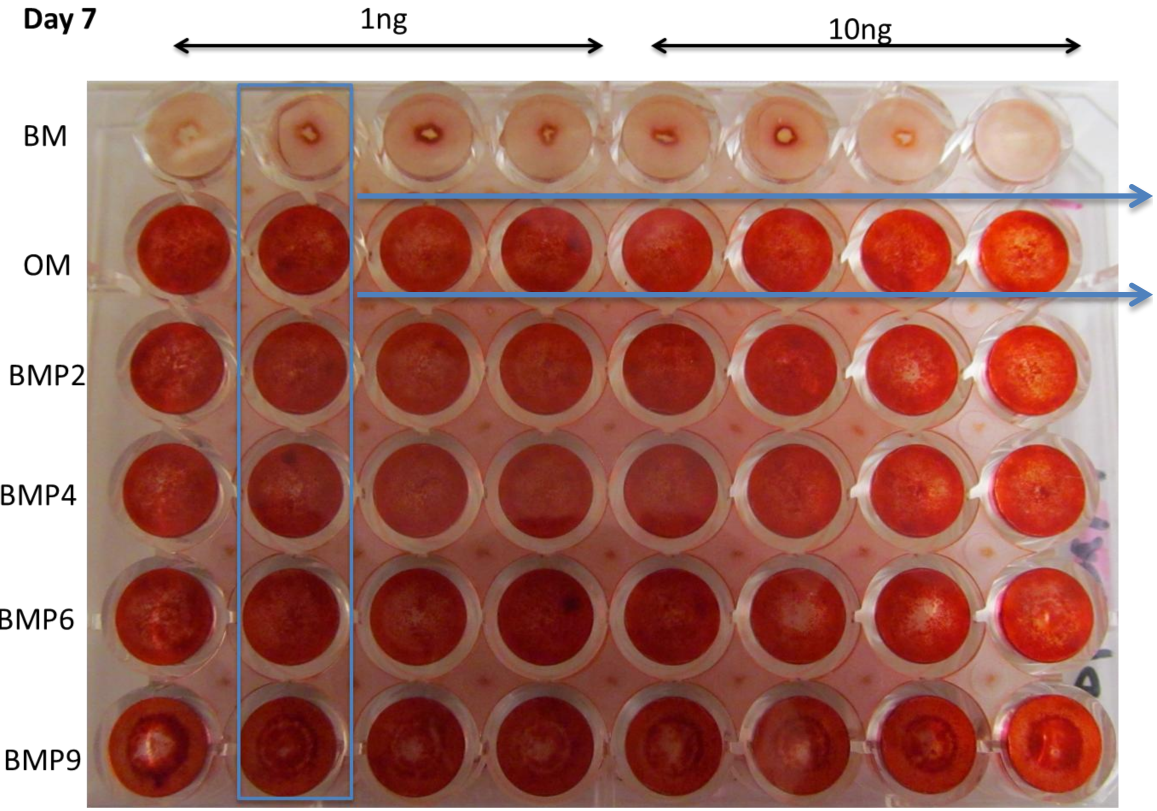

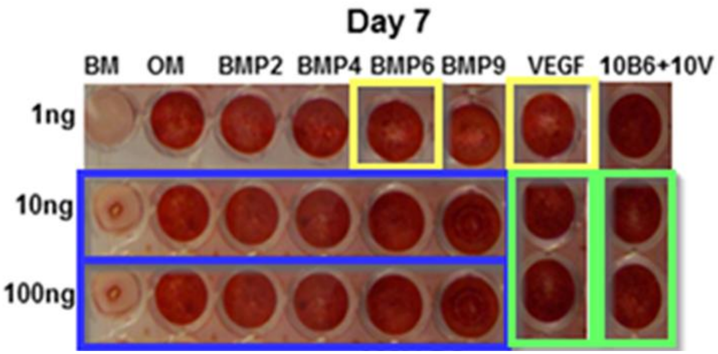

Image of raw data appears on the left, image of published data on the right.

Error 1, is marked in blue:

In figure 1, the Day 7, 1 ng row which is marked with a blue rectangle in the original image was pasted twice as the day 7, 10 ng row as well as the day 7, 100 ng row.

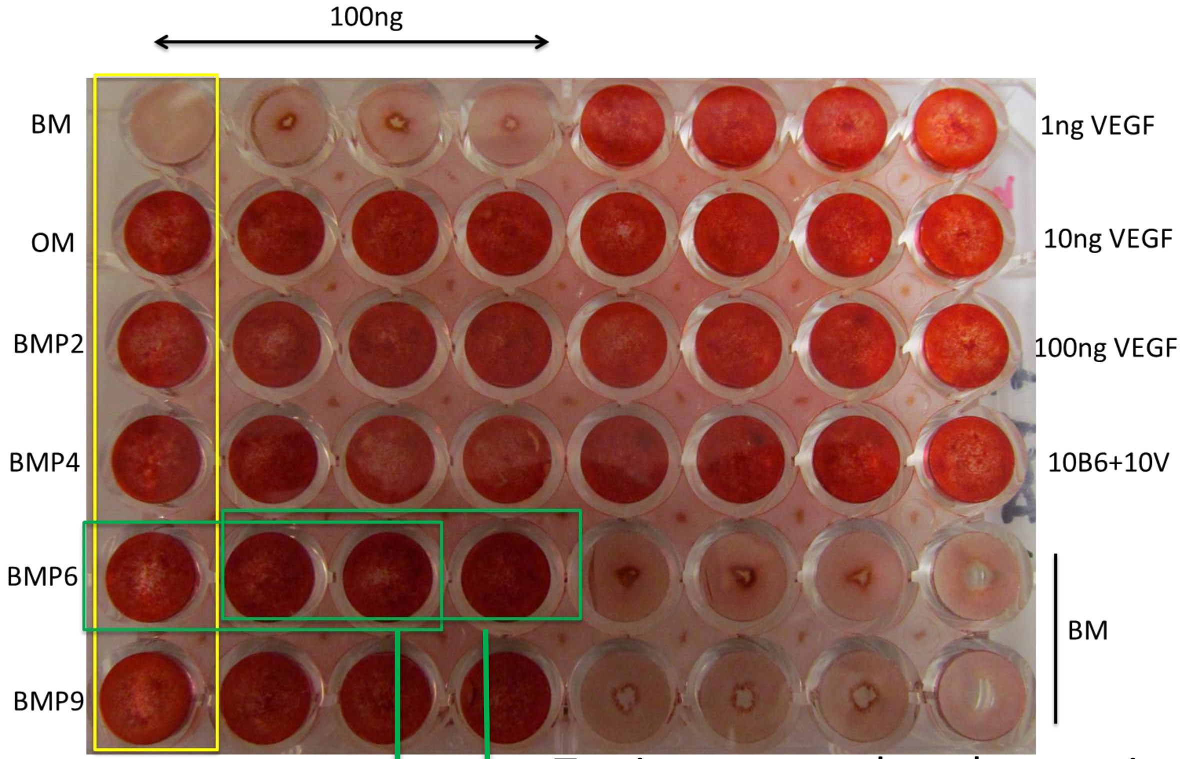

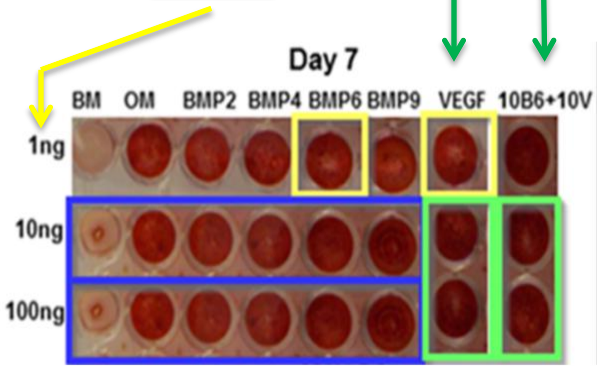

Top image: raw data; bottom image: published data.
Errors 2 and 3, are marked in yellow and in green:

1. The Day 7, 100 ng row marked in yellow in the original image, was pasted as the day 7, 1 ng row.
2. The first 3 wells of the day 7, BMP-6 row, marked in green in the original image, were pasted as the day 7, VEGF row.
3. The last 3 wells of the day 7, BMP-6 row, marked in green in the original image, were pasted as day 7 10B6+10V row.
4. Owing to these mistakes, images of the wells marked in yellow and in green appeared to be similar.

Top image: raw data; bottom image: published data.

Error 4, marked with a pink rectangle:

1. The second, third and fourth well of the BMP-4 row, marked in pink in the original image, were pasted as day 14, VEGF column.
2. The third and fourth well of BMP-4 and the adjoining first well of 10B6+10V, marked with a pink rectangle in the original image, were pasted as day 14, 10B6+10V column.
3. Owing to these mistakes, the wells marked with a pink rectangles in the image, appeared to be similar.

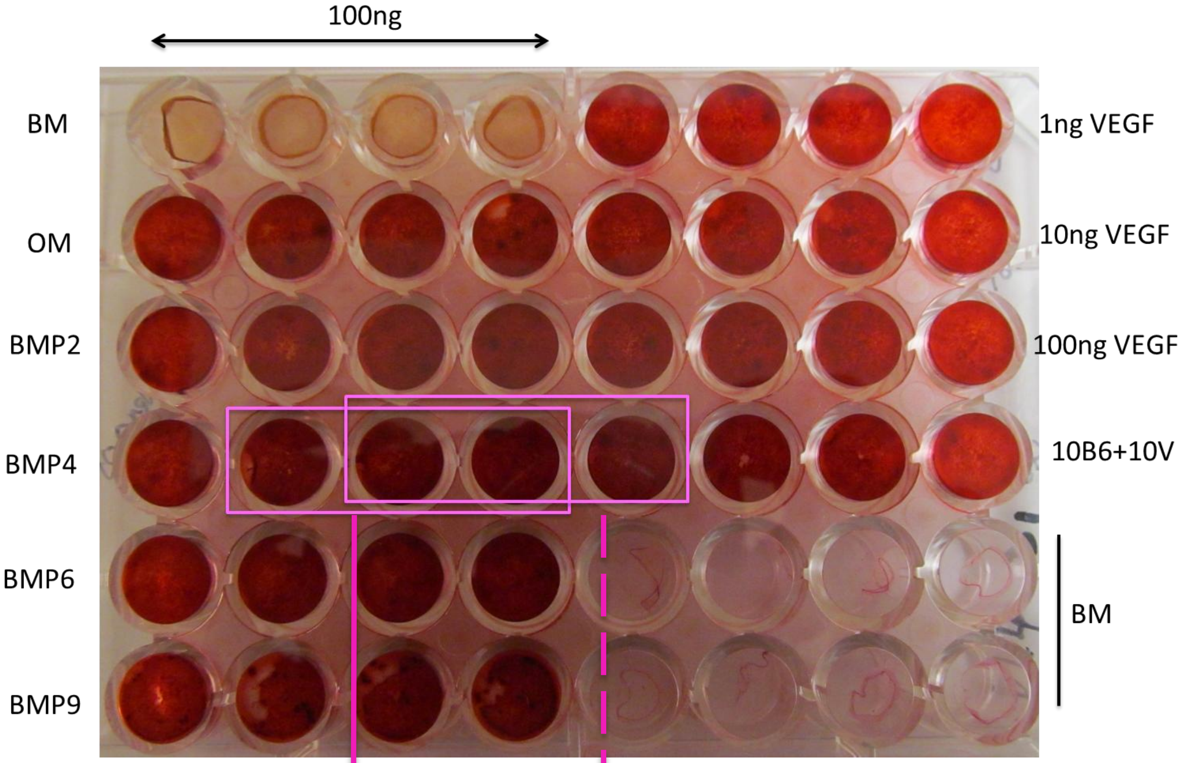

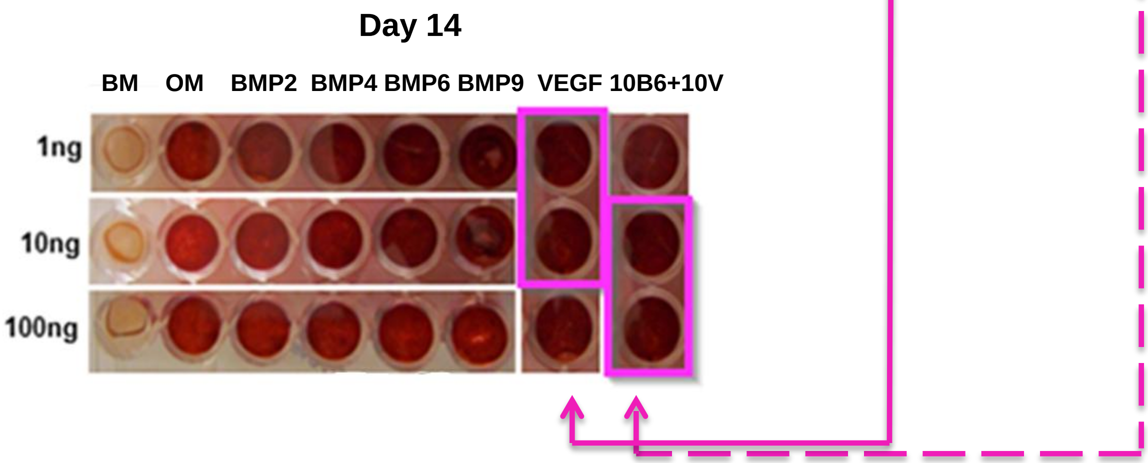

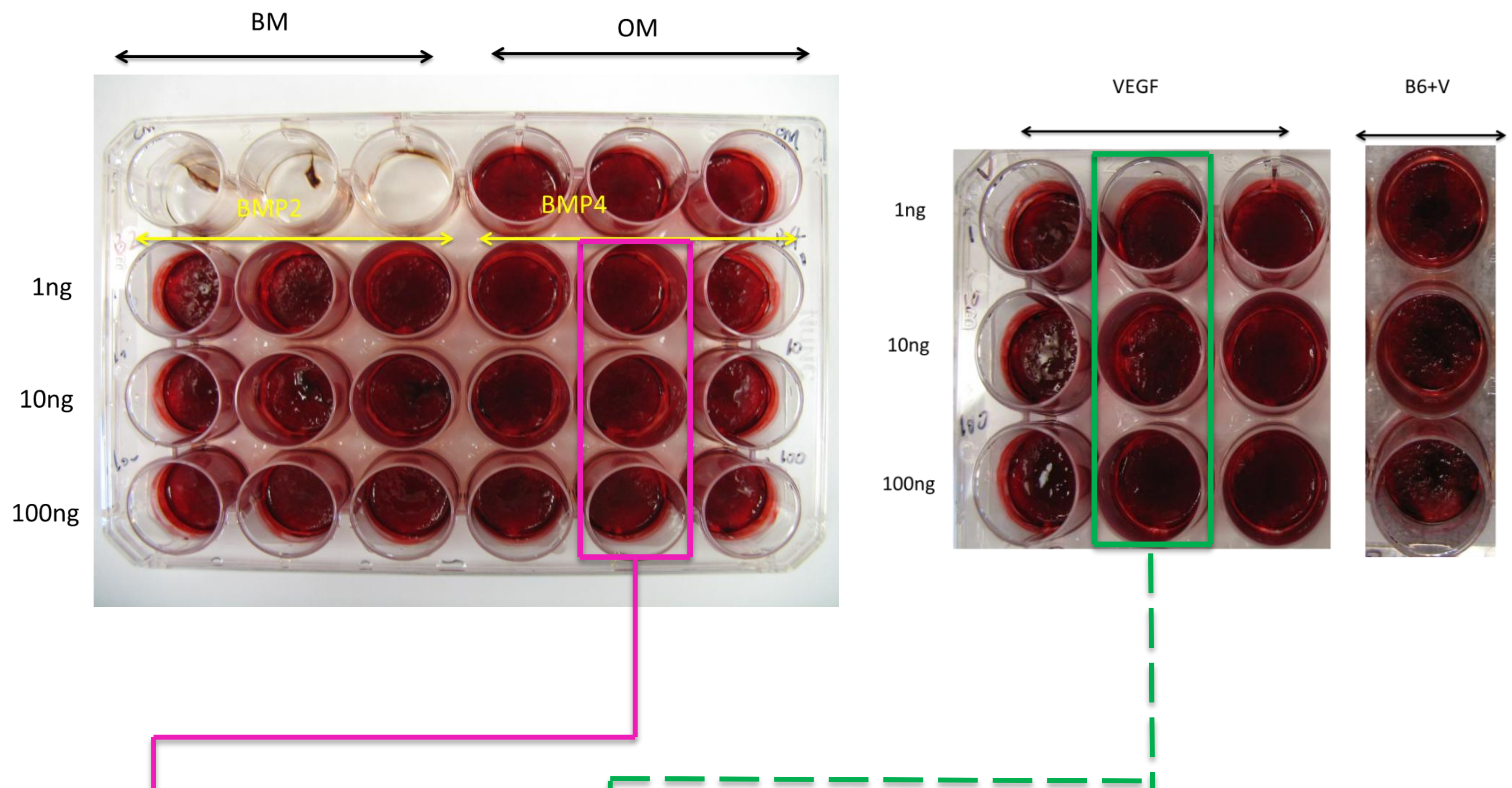

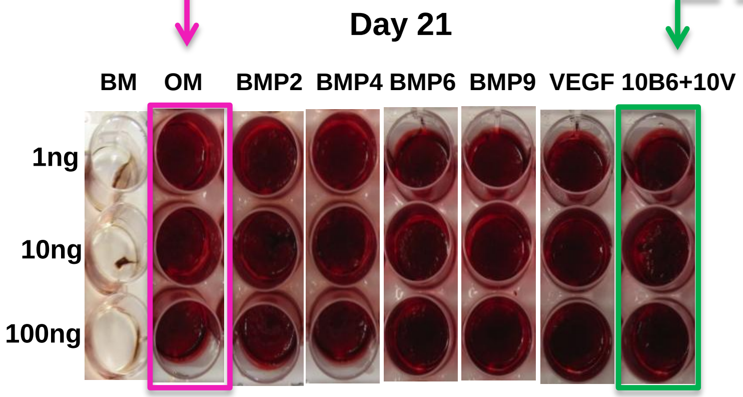

Top images: raw data; bottom image: published data.

Error 5, marked with pink and green rectangles

1. In the Day 21 panel, the 2nd column of BMP4 wells in the original image, marked in pink, was pasted as OM.

2. The 2nd column of VEGF in the original image, indicated with a green rectangle was pasted as 10B6+10V.
