# Supplementary figures and images for: Correction: BMP-Non-Responsive Sca1+CD73+CD44+ Mouse Bone Marrow Derived Osteoprogenitor Cells Respond to Combination of VEGF and BMP-6 to Display Enhanced Osteoblastic Differentiation and Ectopic Bone Formation
Source: PLoS One. 2019 Jan 31;14(1):e0211782. doi: 10.1371/journal.pone.0211782 (PMC6355026; doi:10.1371/journal.pone.0211782)

## Slide 1
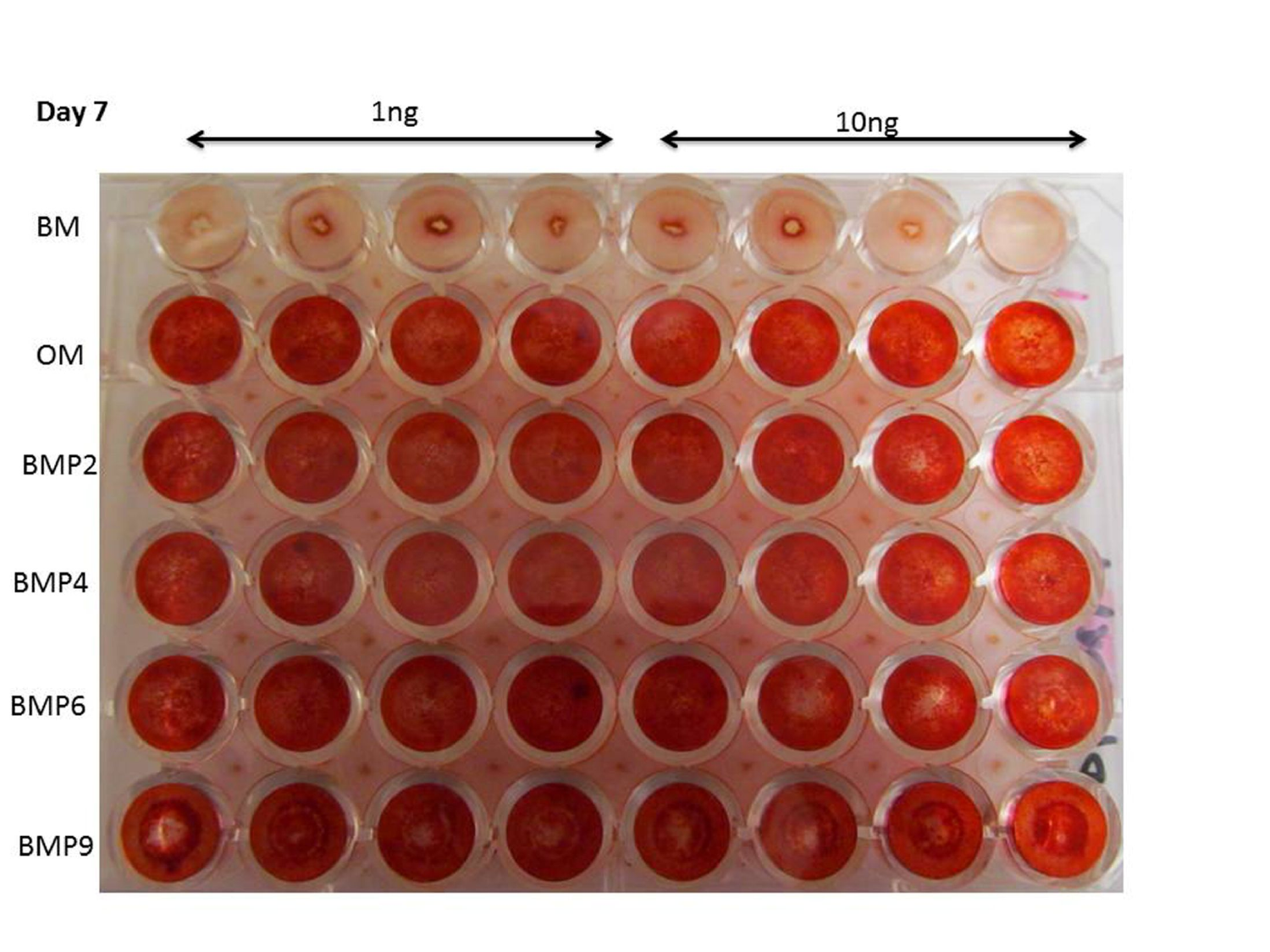

## Slide 2
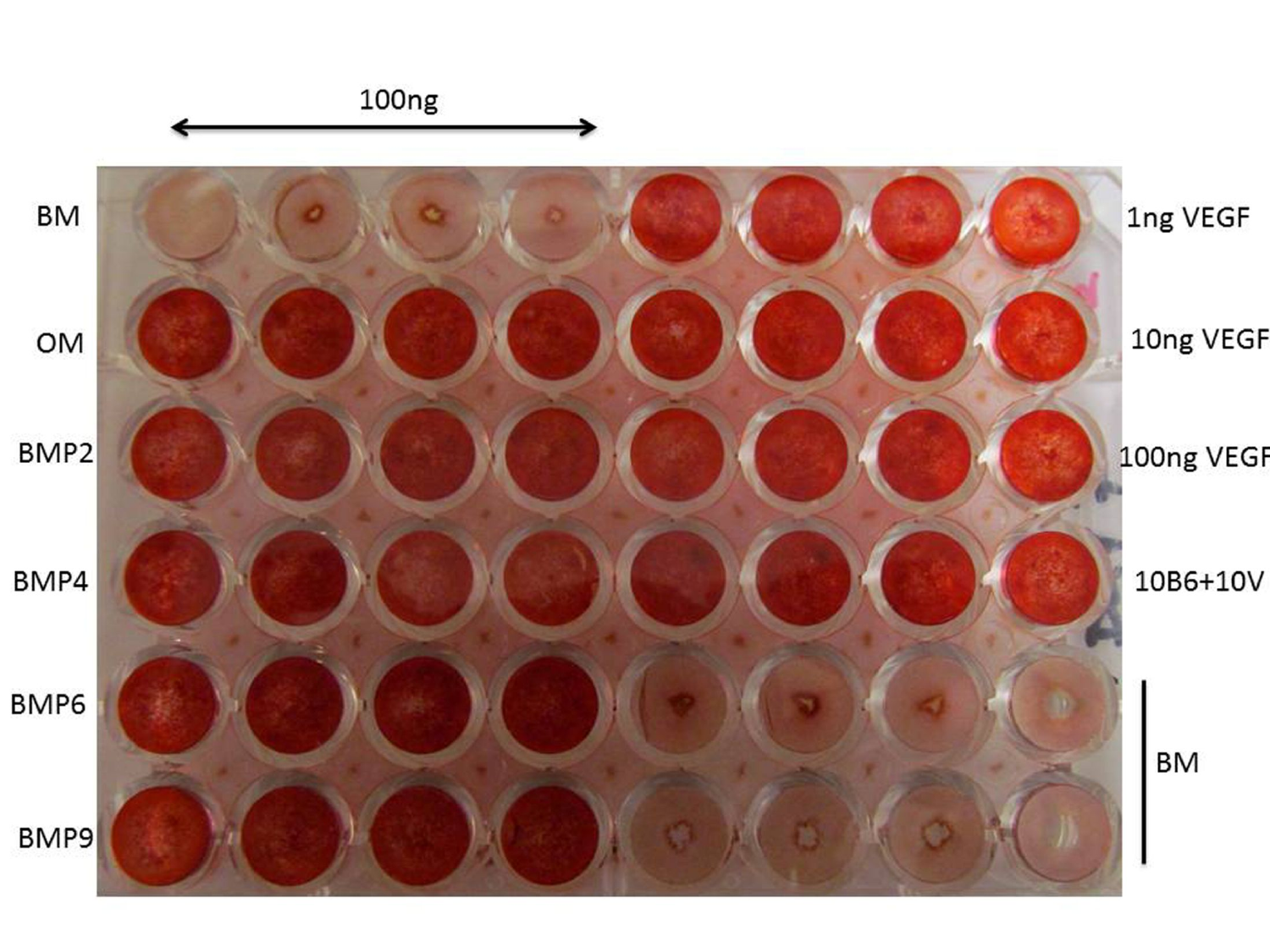

## Slide 3
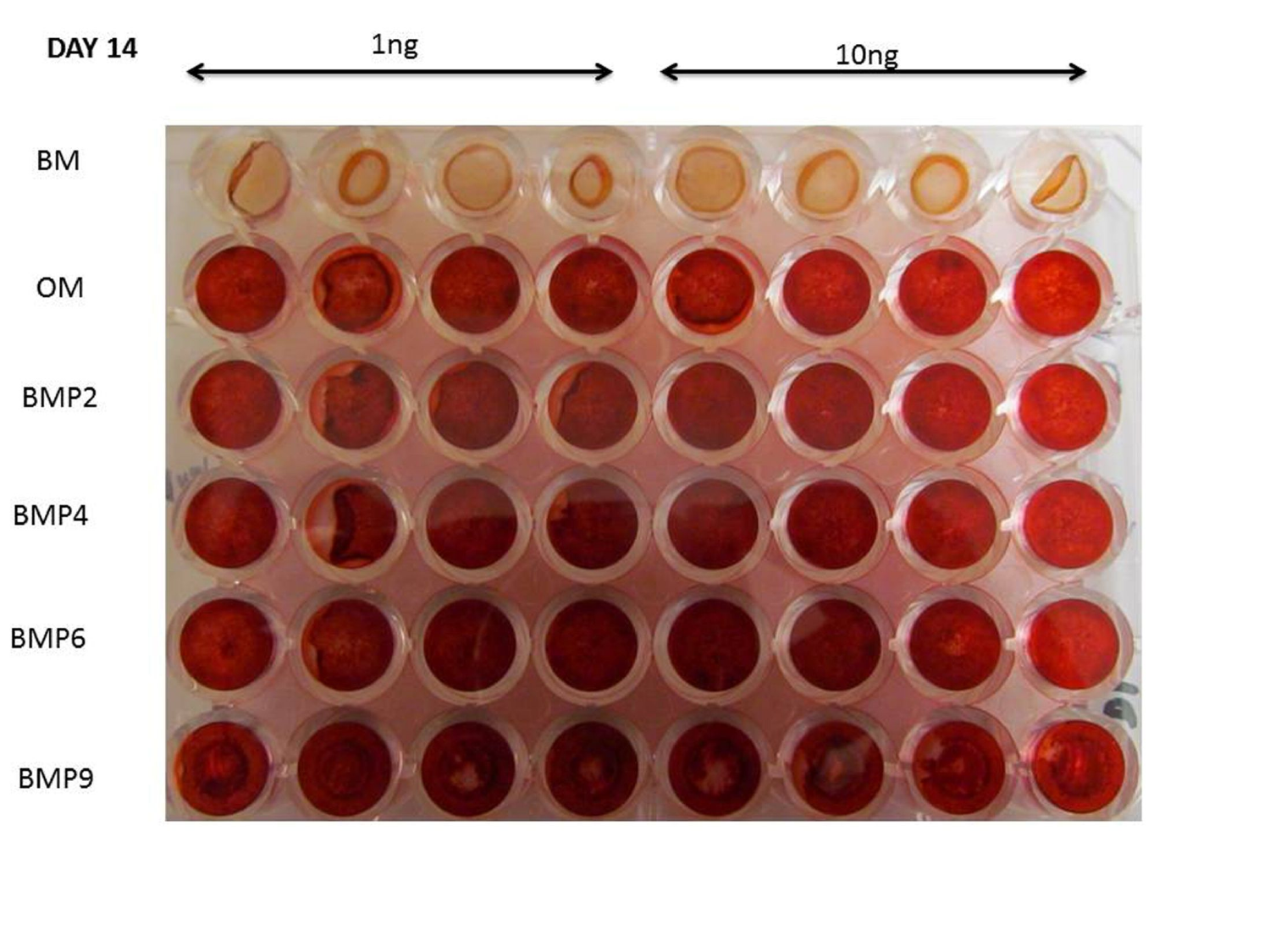

## Slide 4
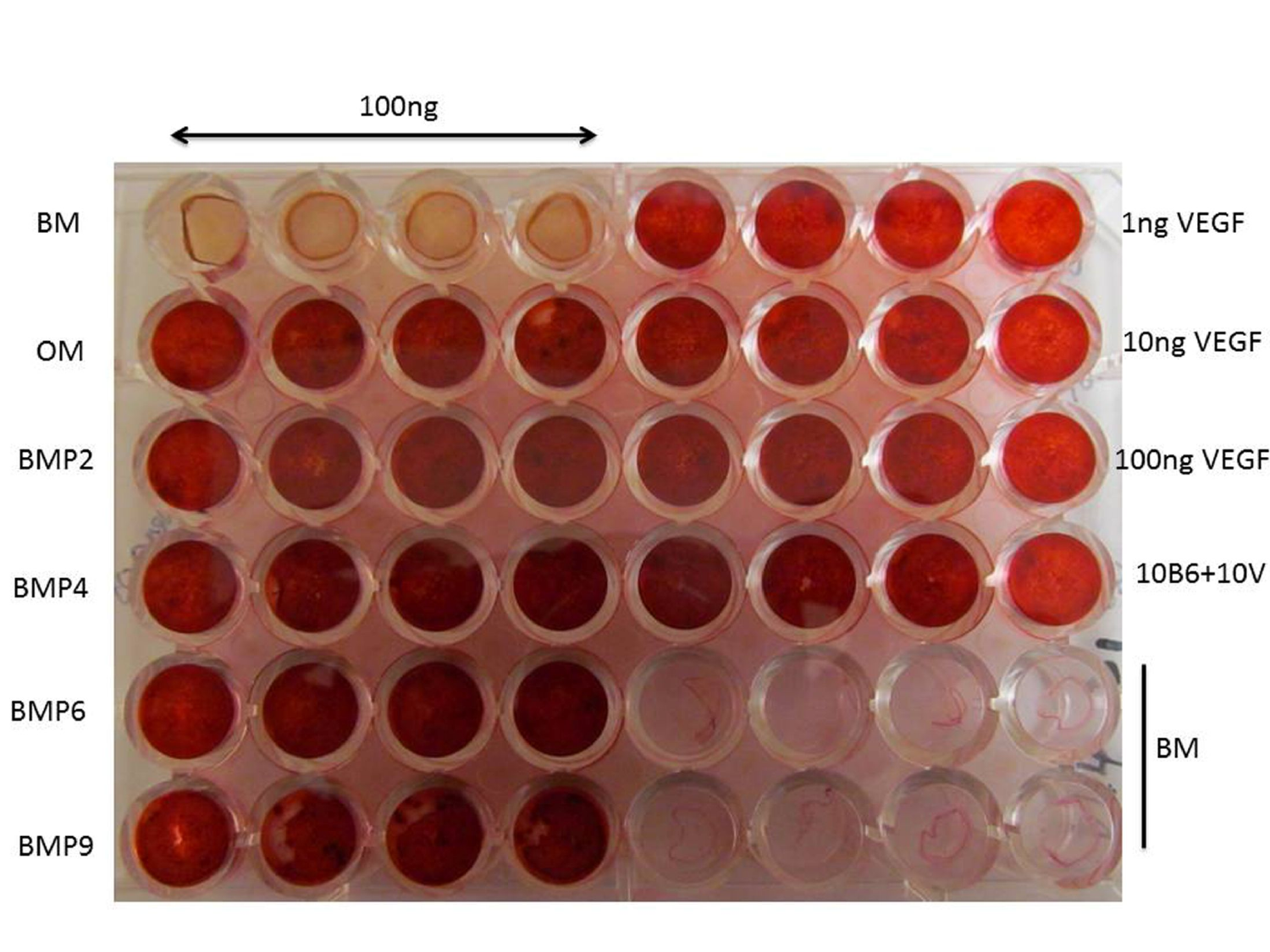

## Slide 5
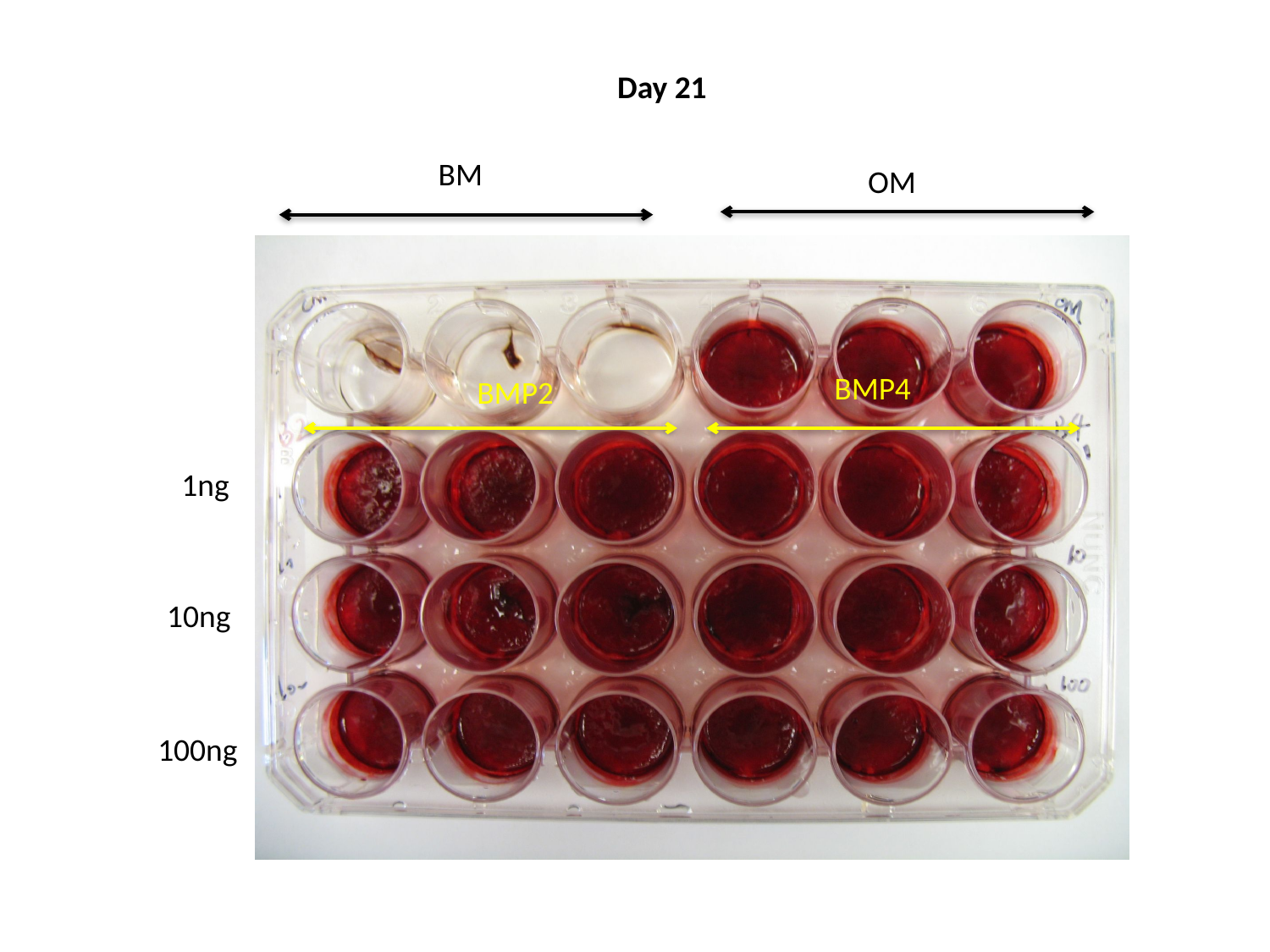

Day 21
BM
OM
BMP4
BMP2
1ng
10ng
100ng

## Slide 6
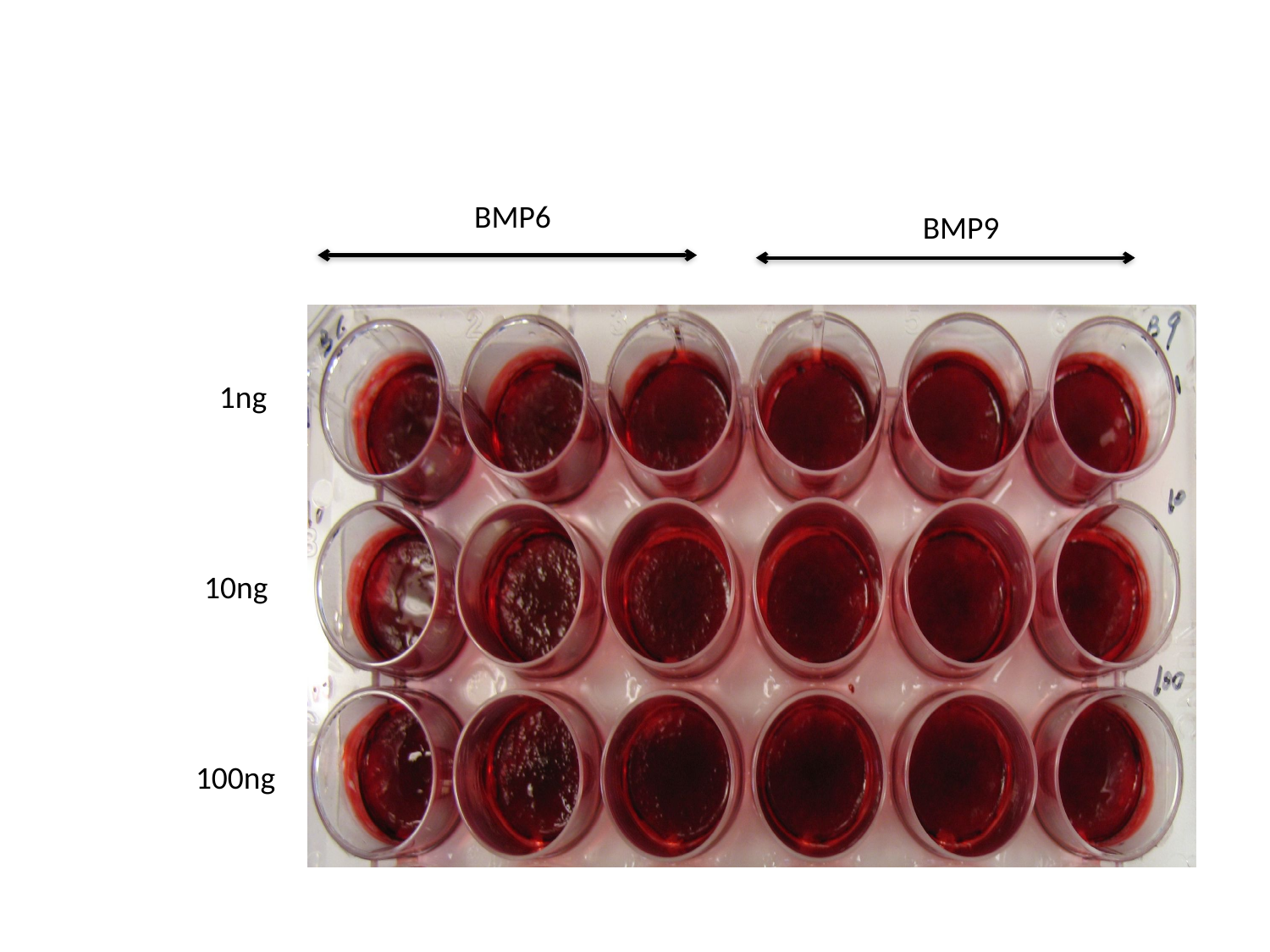

BMP6
BMP9
1ng
10ng
100ng

## Slide 7
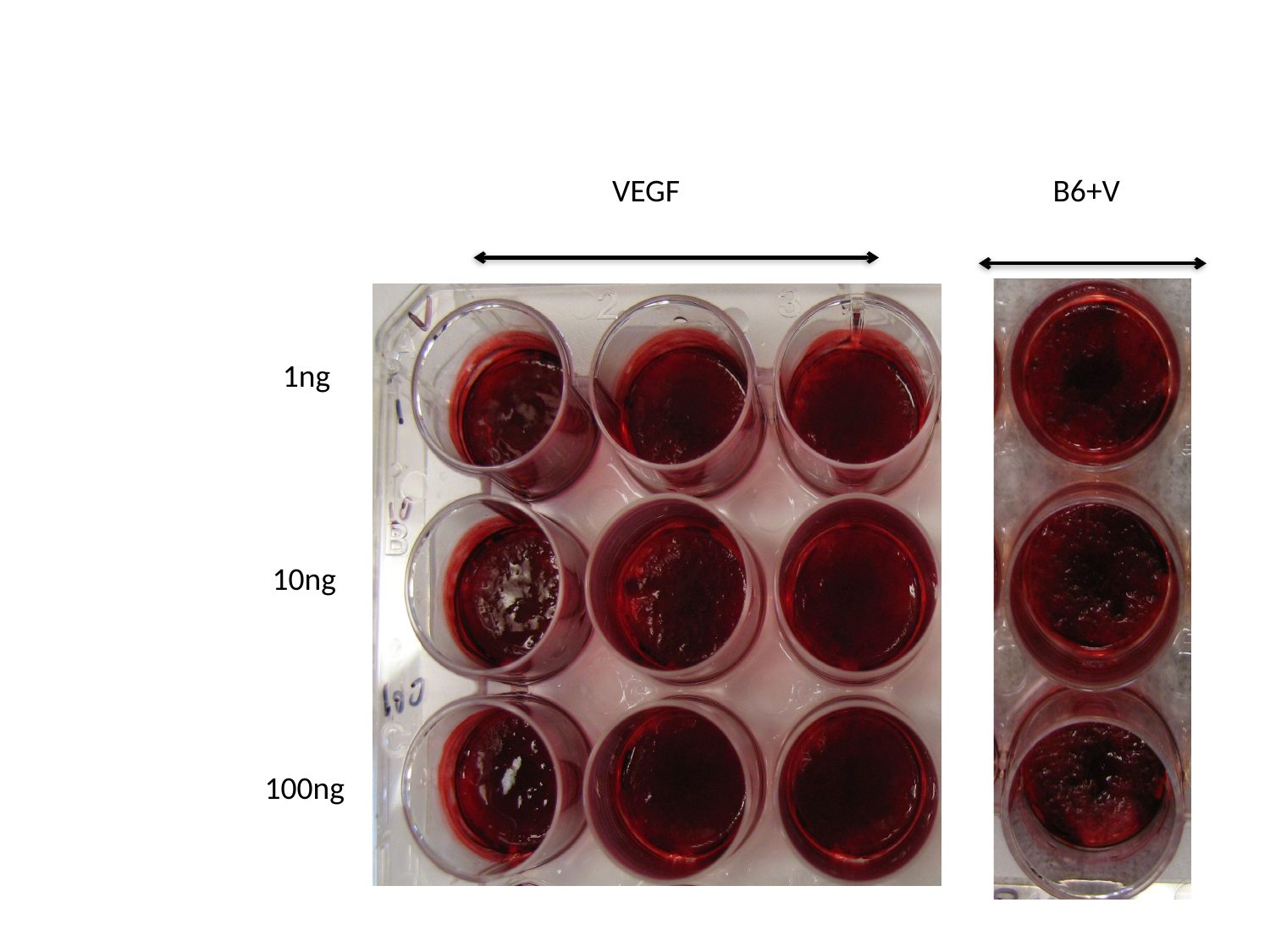

VEGF
B6+V
1ng
10ng
100ng

Supplement: S1 Data — (ZIP) [file pone.0211782.s002.zip › Figure1Images.pptx]

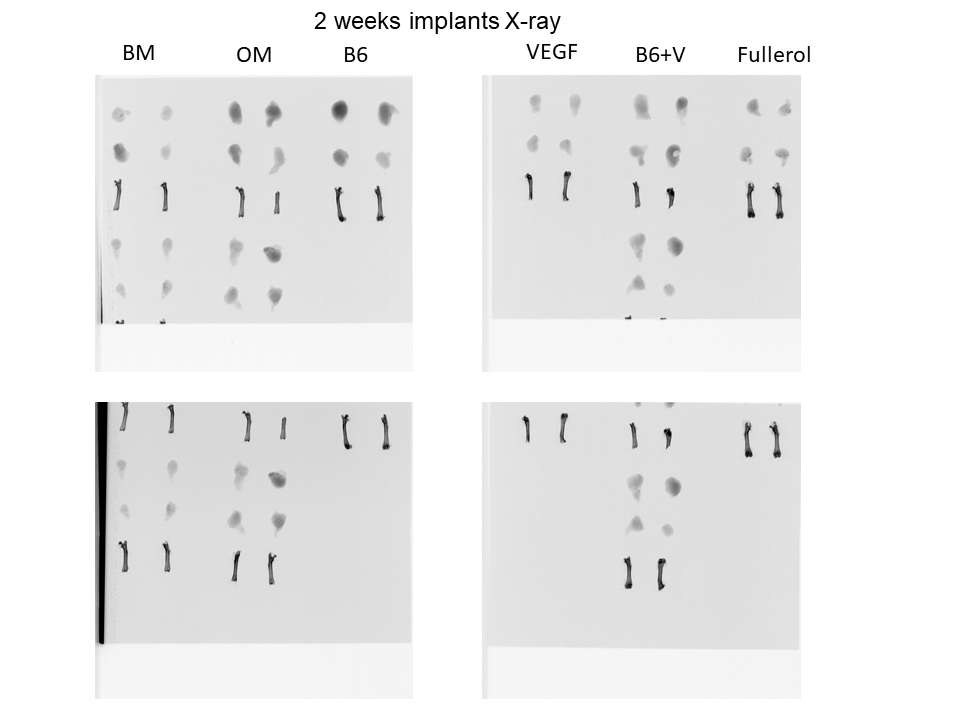

Supplement: S7 Data — (ZIP) [file pone.0211782.s008.zip › 2 weeks implants X-ray.TIF]

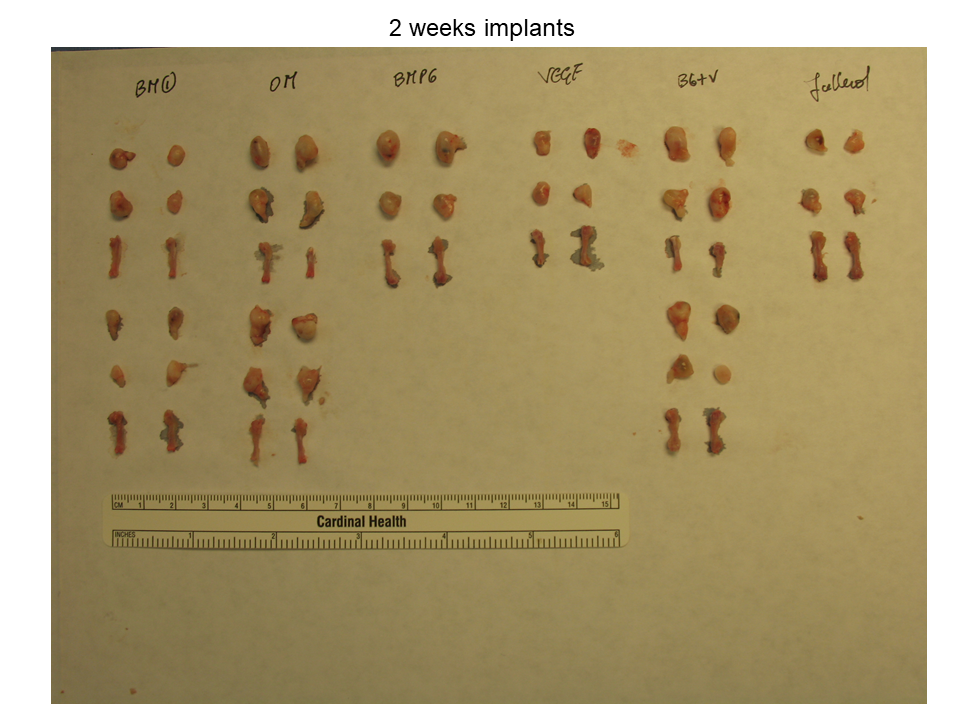

Supplement: S7 Data — (ZIP) [file pone.0211782.s008.zip › 2 weeks implants.TIF]

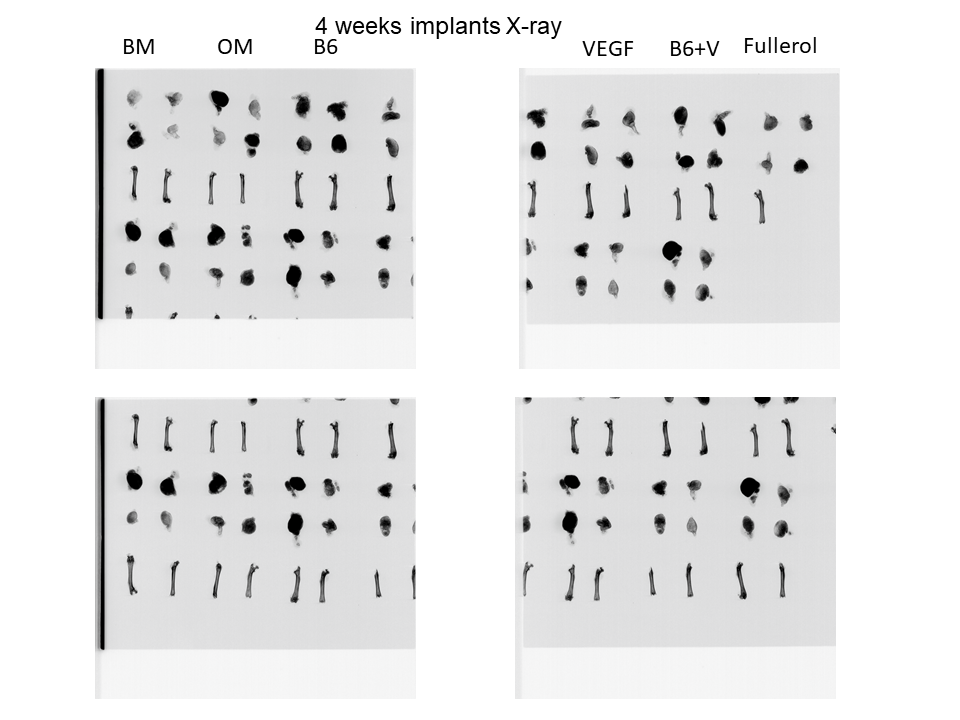

Supplement: S7 Data — (ZIP) [file pone.0211782.s008.zip › 4 weeks implants X-ray.TIF]

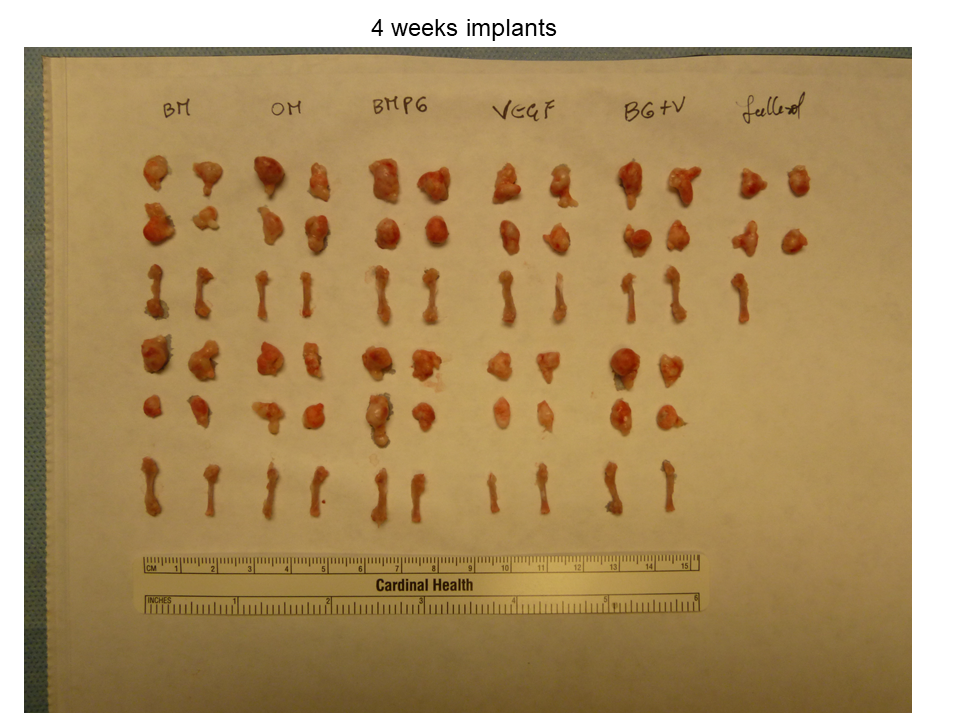

Supplement: S7 Data — (ZIP) [file pone.0211782.s008.zip › 4 weeks implants.TIF]

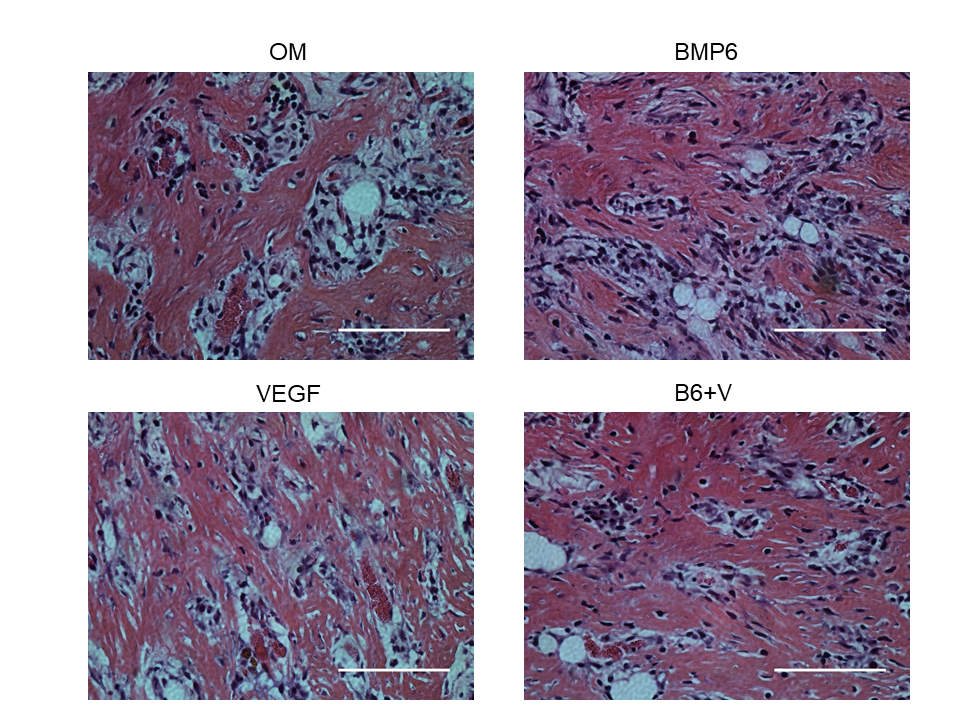

Supplement: S7 Data — (ZIP) [file pone.0211782.s008.zip › Fig6E.TIF]
