# Supplementary material for: Correction: BMP-Non-Responsive Sca1+CD73+CD44+ Mouse Bone Marrow Derived Osteoprogenitor Cells Respond to Combination of VEGF and BMP-6 to Display Enhanced Osteoblastic Differentiation and Ectopic Bone Formation
Source: PLoS One. 2019 Jan 31;14(1):e0211782. doi: 10.1371/journal.pone.0211782 (PMC6355026; doi:10.1371/journal.pone.0211782)
Supplement: S2 Data — (ZIP) [file pone.0211782.s003.zip › Figure2Images.pptx]

## Slide 1
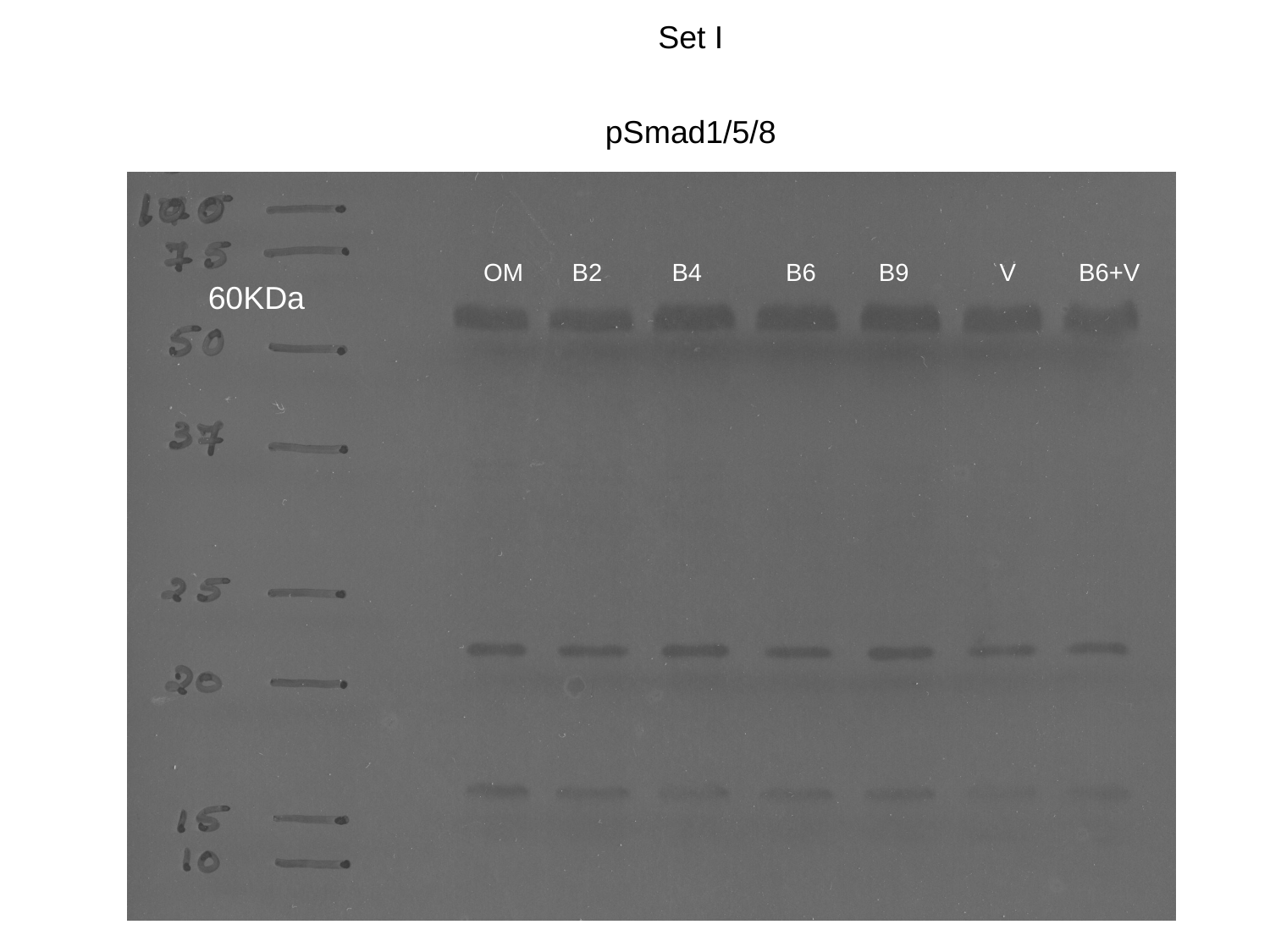

Set I
OM B2 B4 B6 B9 V B6+V
60KDa
pSmad1/5/8

## Slide 2
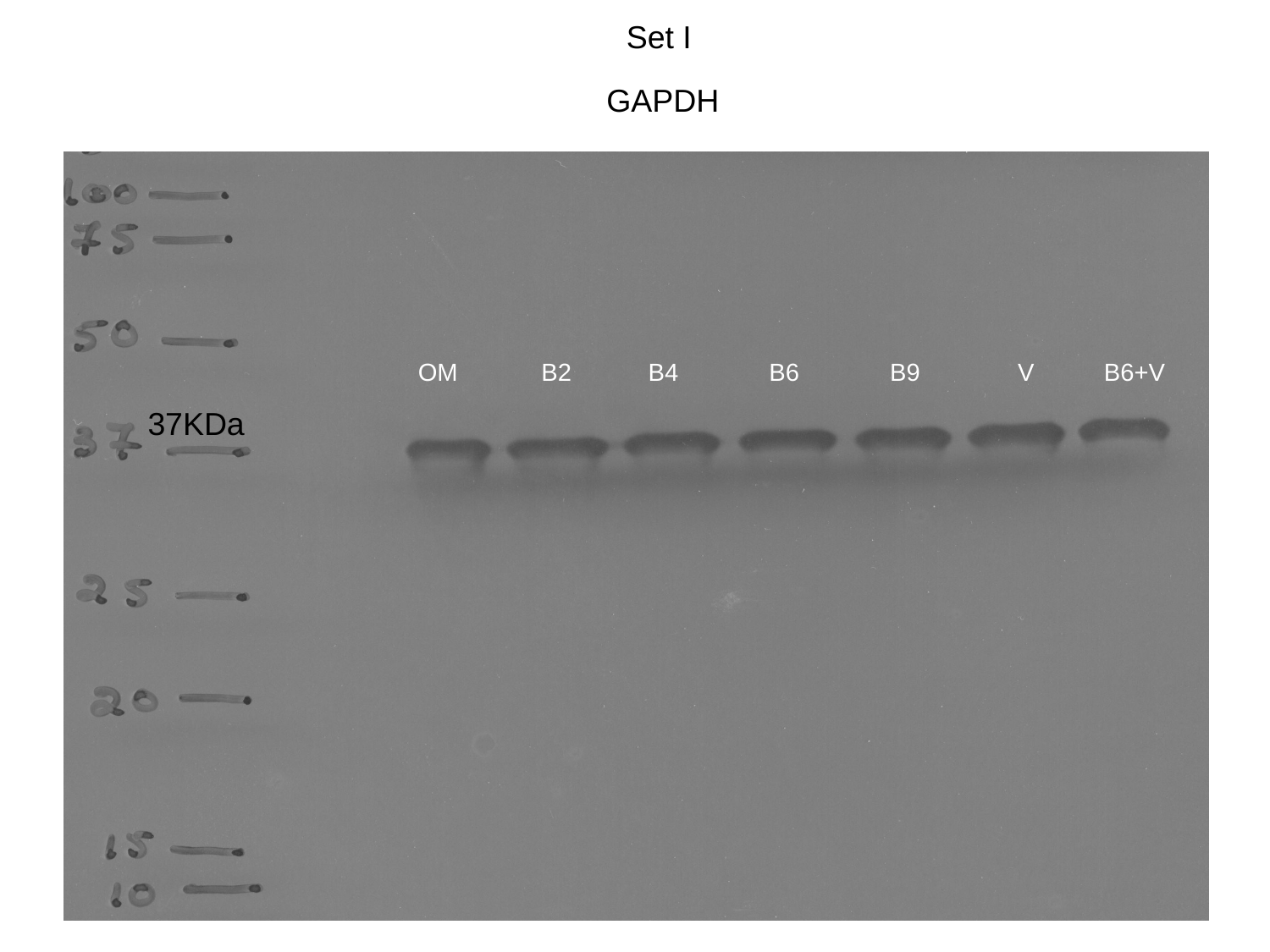

Set I
 OM B2 B4 B6 B9 V B6+V
37KDa
GAPDH

## Slide 3
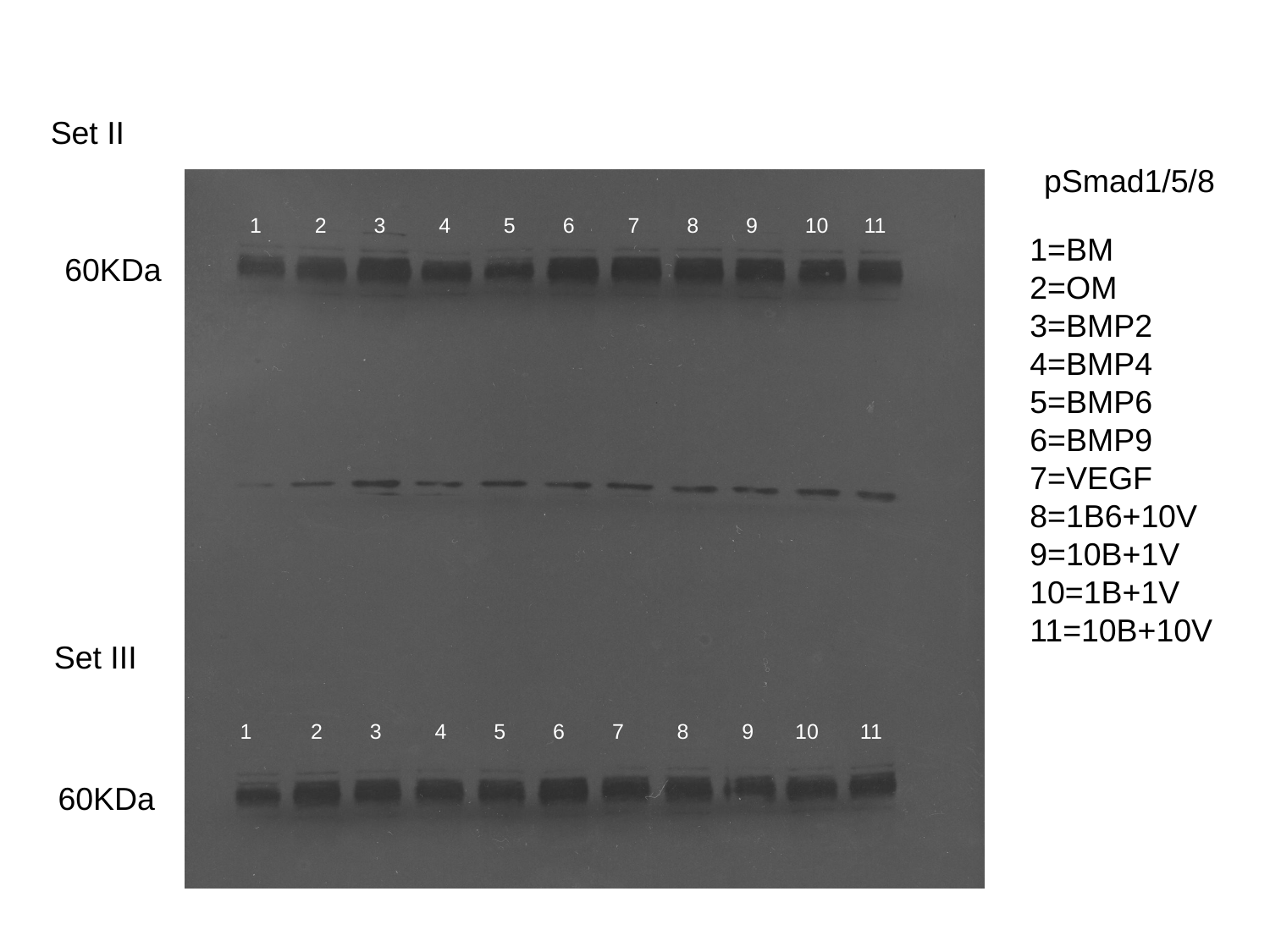

Set II
 1 2 3 4 5 6 7 8 9 10 11
60KDa
Set III
 1 2 3 4 5 6 7 8 9 10 11
60KDa
pSmad1/5/8
1=BM
2=OM
3=BMP2
4=BMP4
5=BMP6
6=BMP9
7=VEGF
8=1B6+10V
9=10B+1V
10=1B+1V
11=10B+10V

## Slide 4
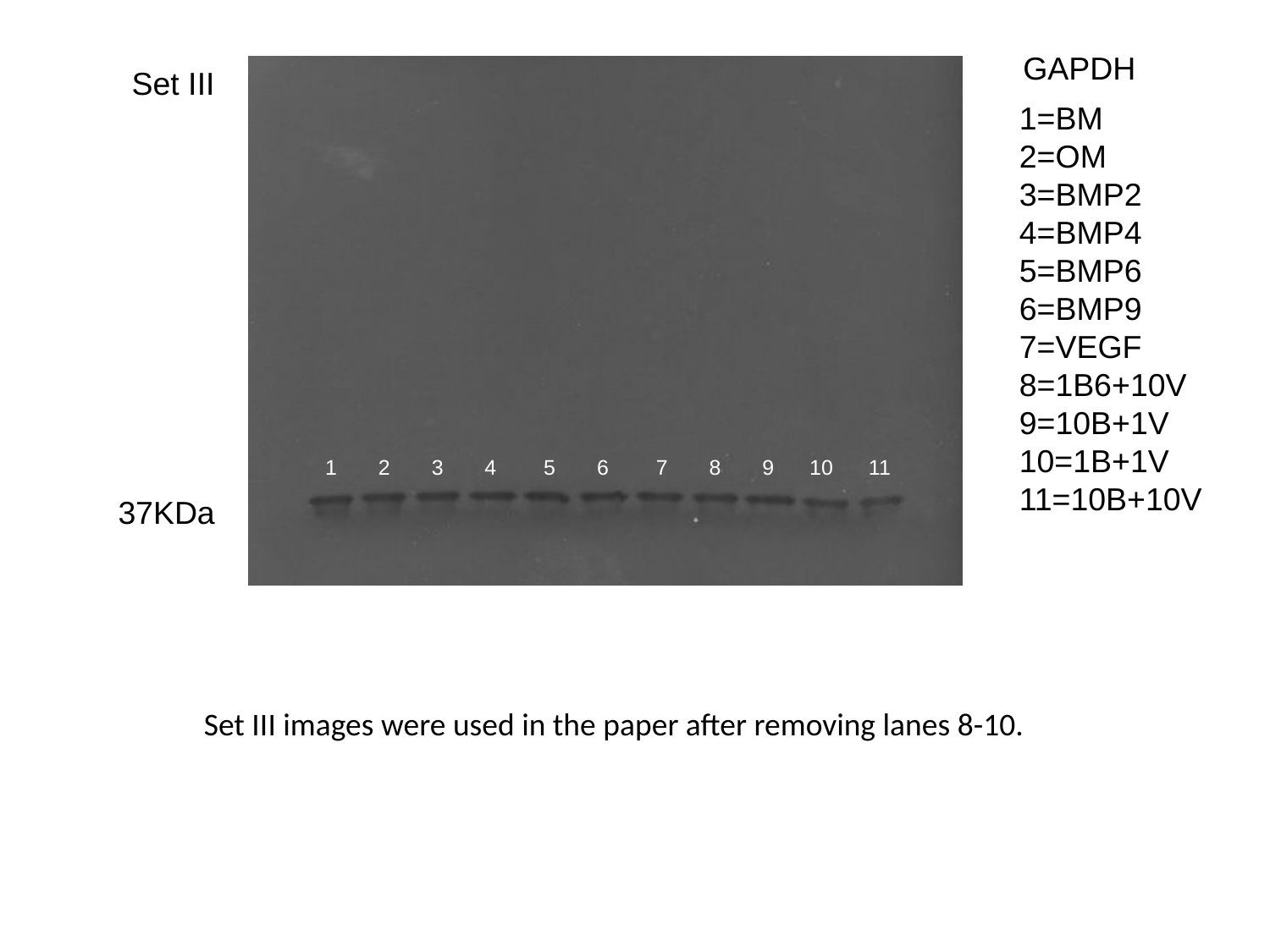

GAPDH
Set III
37KDa
1=BM
2=OM
3=BMP2
4=BMP4
5=BMP6
6=BMP9
7=VEGF
8=1B6+10V
9=10B+1V
10=1B+1V
11=10B+10V
 1 2 3 4 5 6 7 8 9 10 11
 1 2 3 4 5 6 7 8 9 10 11
Set III images were used in the paper after removing lanes 8-10.
