# Supplementary material for: Correction: BMP-Non-Responsive Sca1+CD73+CD44+ Mouse Bone Marrow Derived Osteoprogenitor Cells Respond to Combination of VEGF and BMP-6 to Display Enhanced Osteoblastic Differentiation and Ectopic Bone Formation
Source: PLoS One. 2019 Jan 31;14(1):e0211782. doi: 10.1371/journal.pone.0211782 (PMC6355026; doi:10.1371/journal.pone.0211782)
Supplement: S3 Data — (PPTX) [file pone.0211782.s004.pptx]

## Slide 1
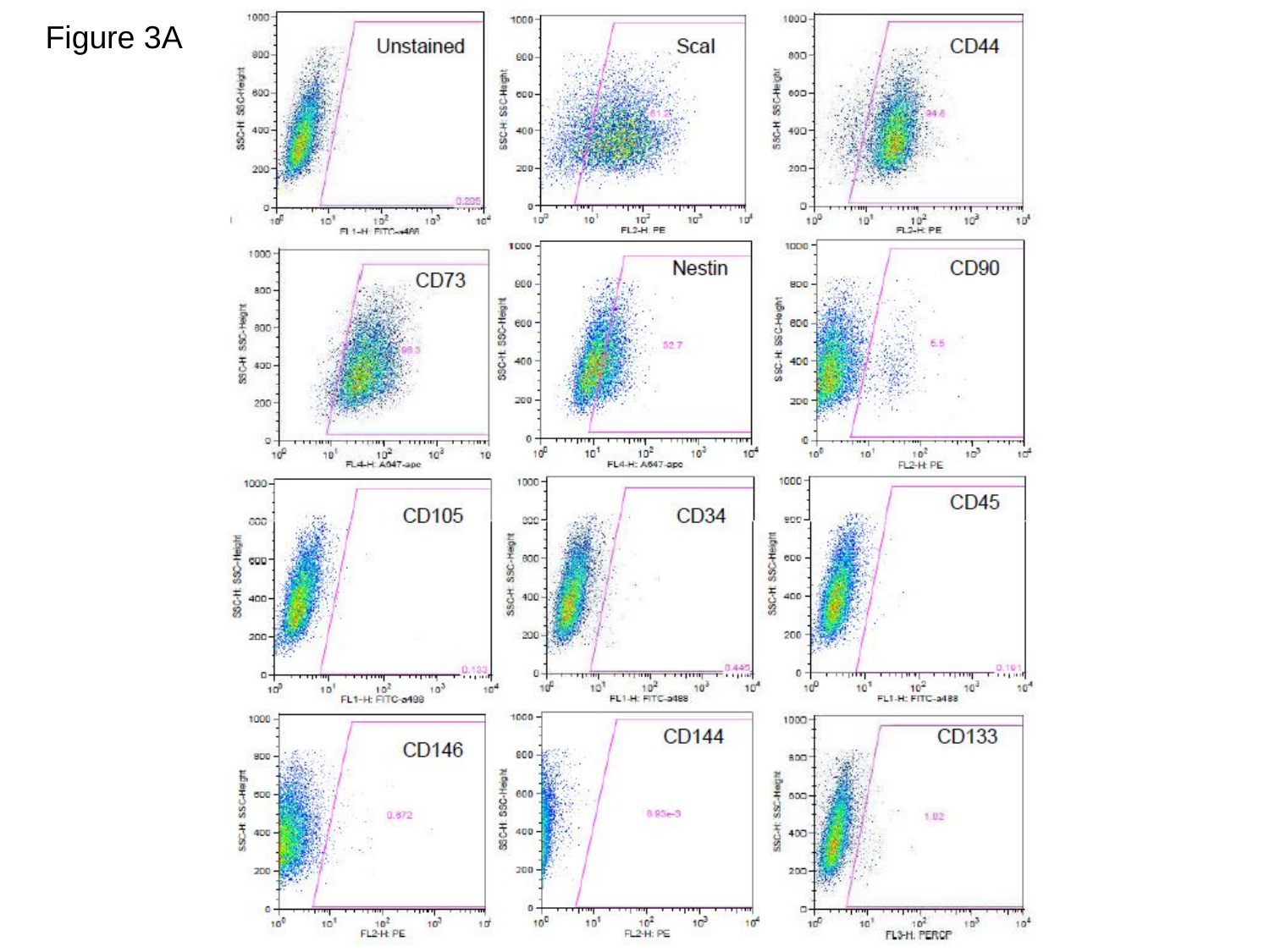

Figure 3A

## Slide 2
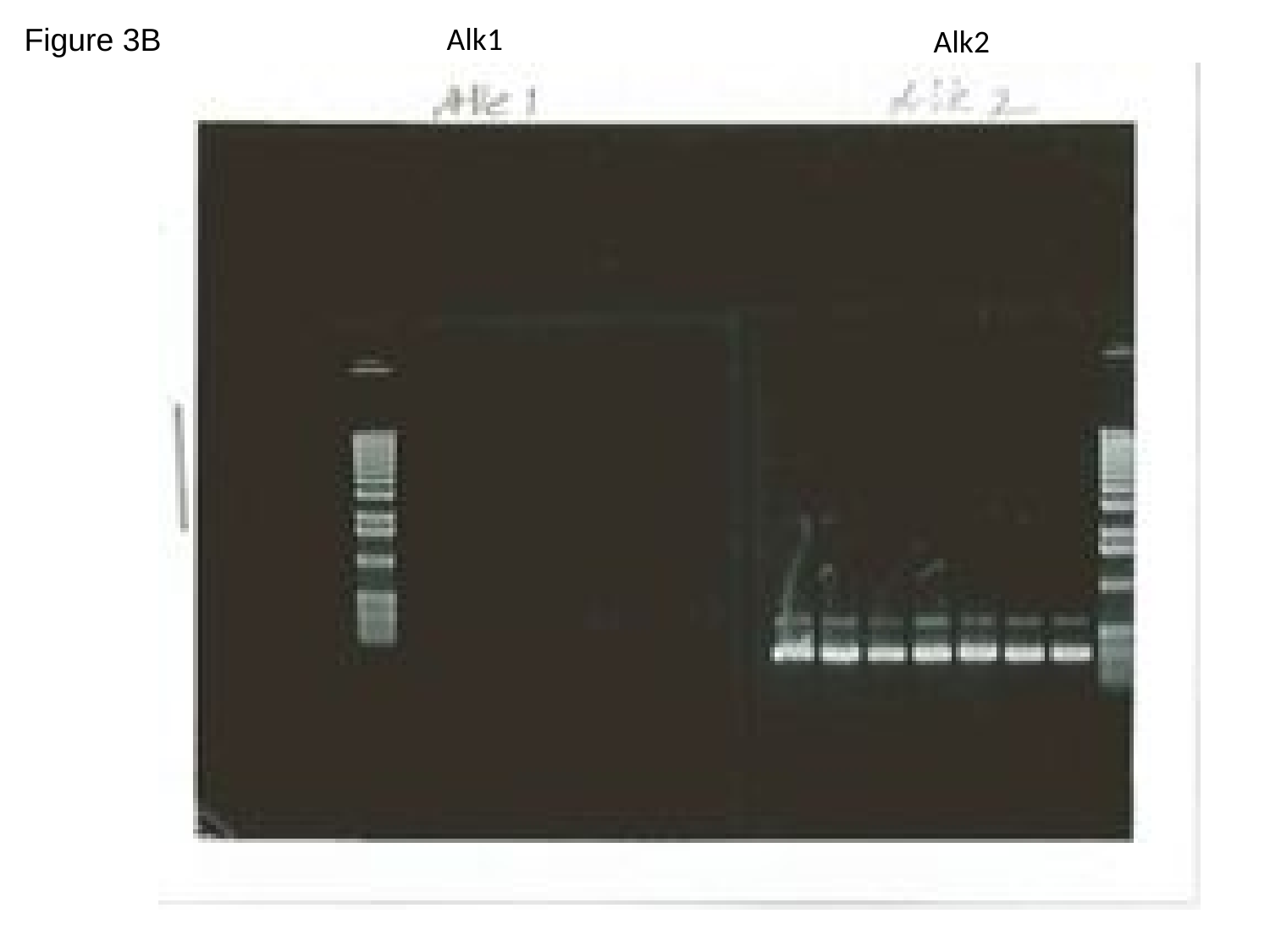

Alk1
Figure 3B
Alk2

## Slide 3
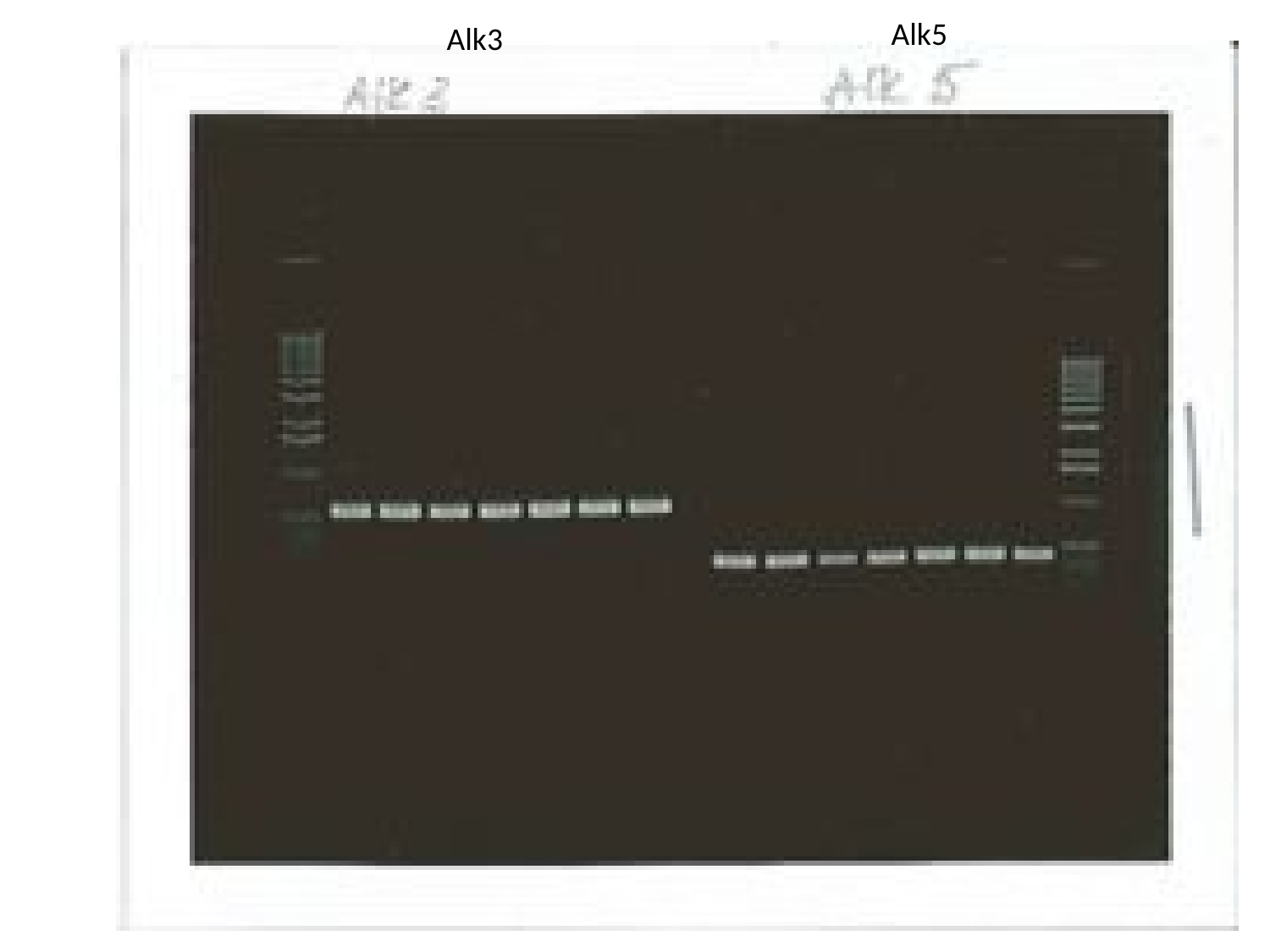

Alk5
Alk3

## Slide 4
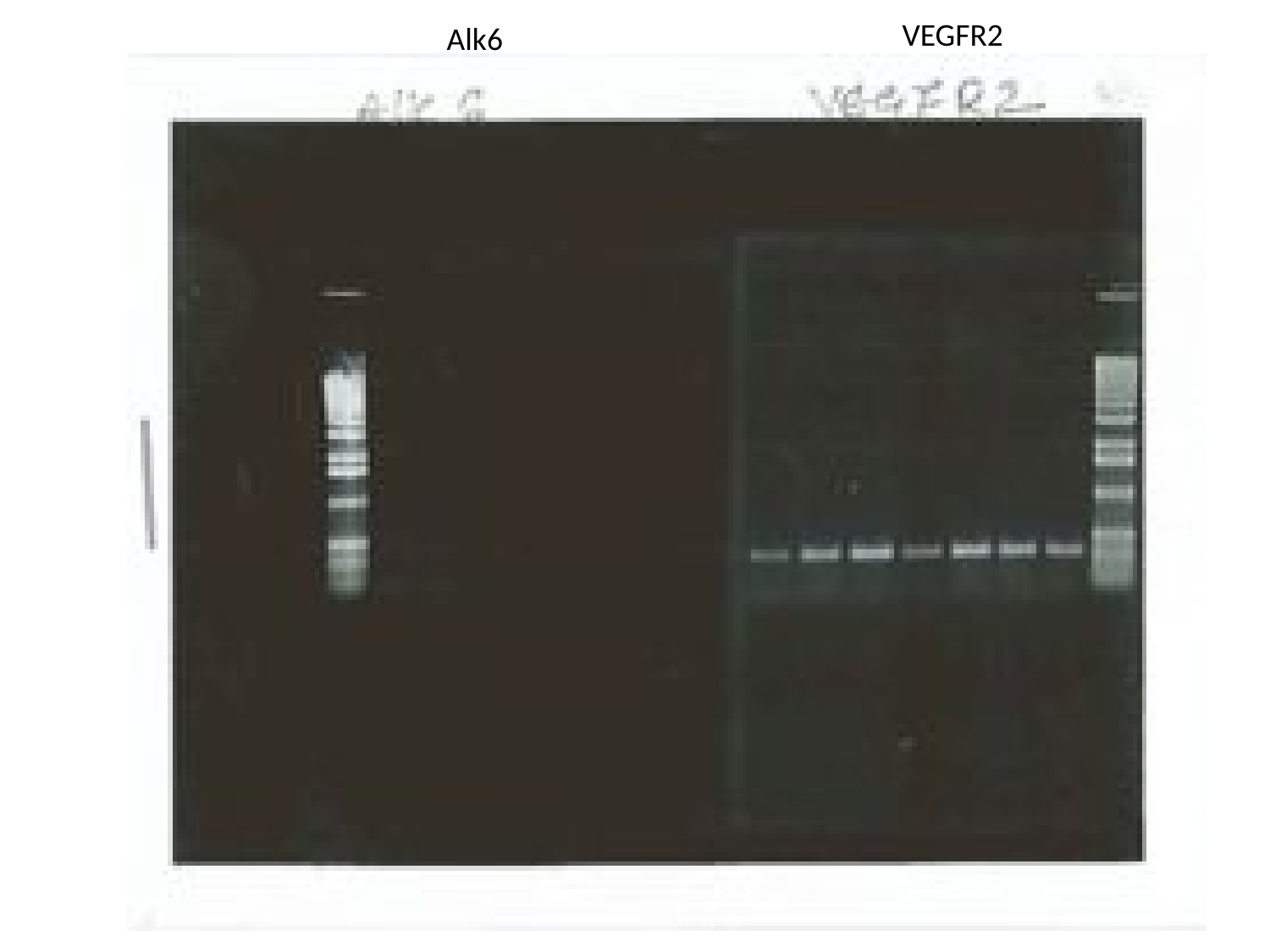

VEGFR2
Alk6

## Slide 5
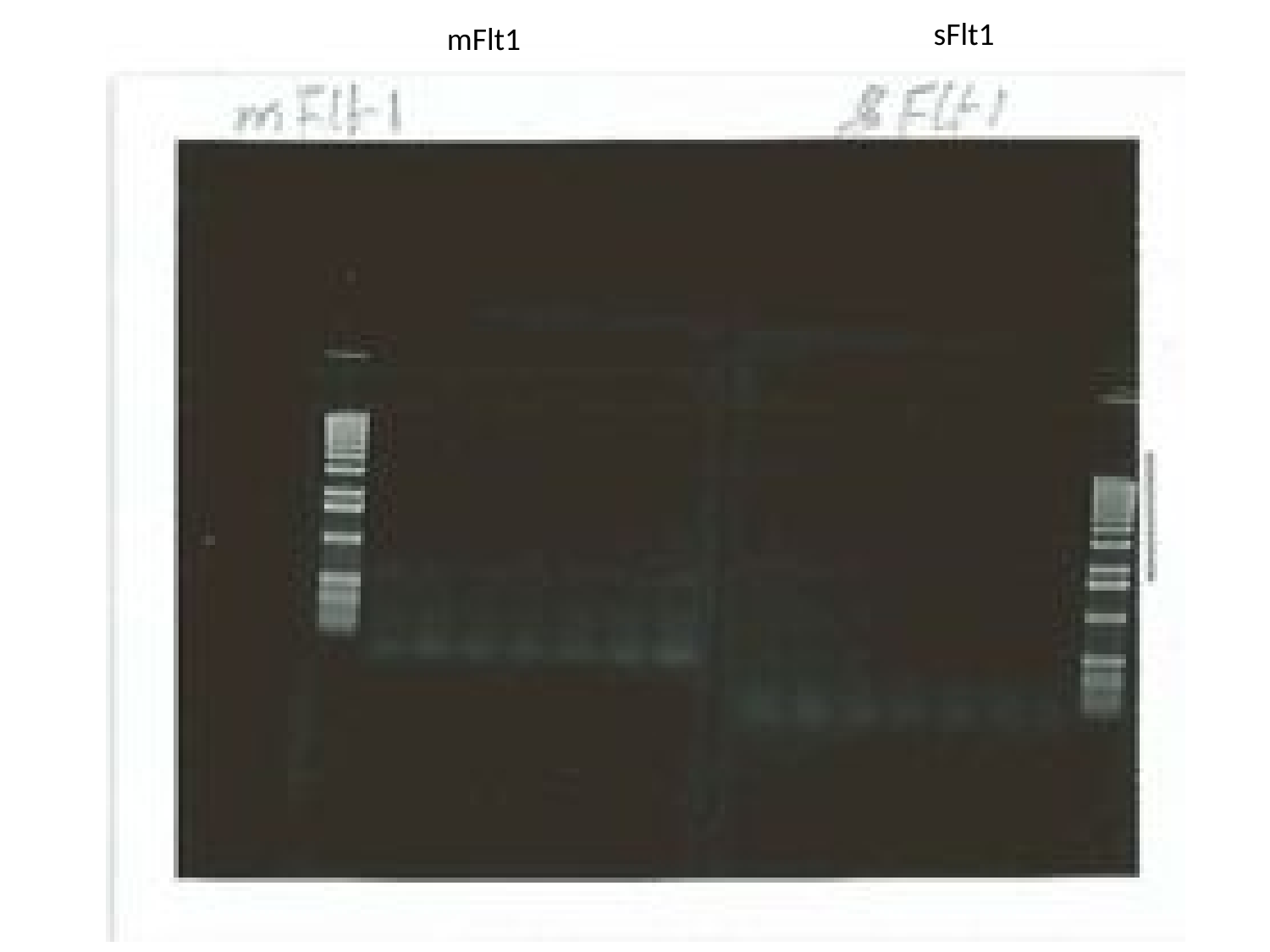

sFlt1
mFlt1

## Slide 6
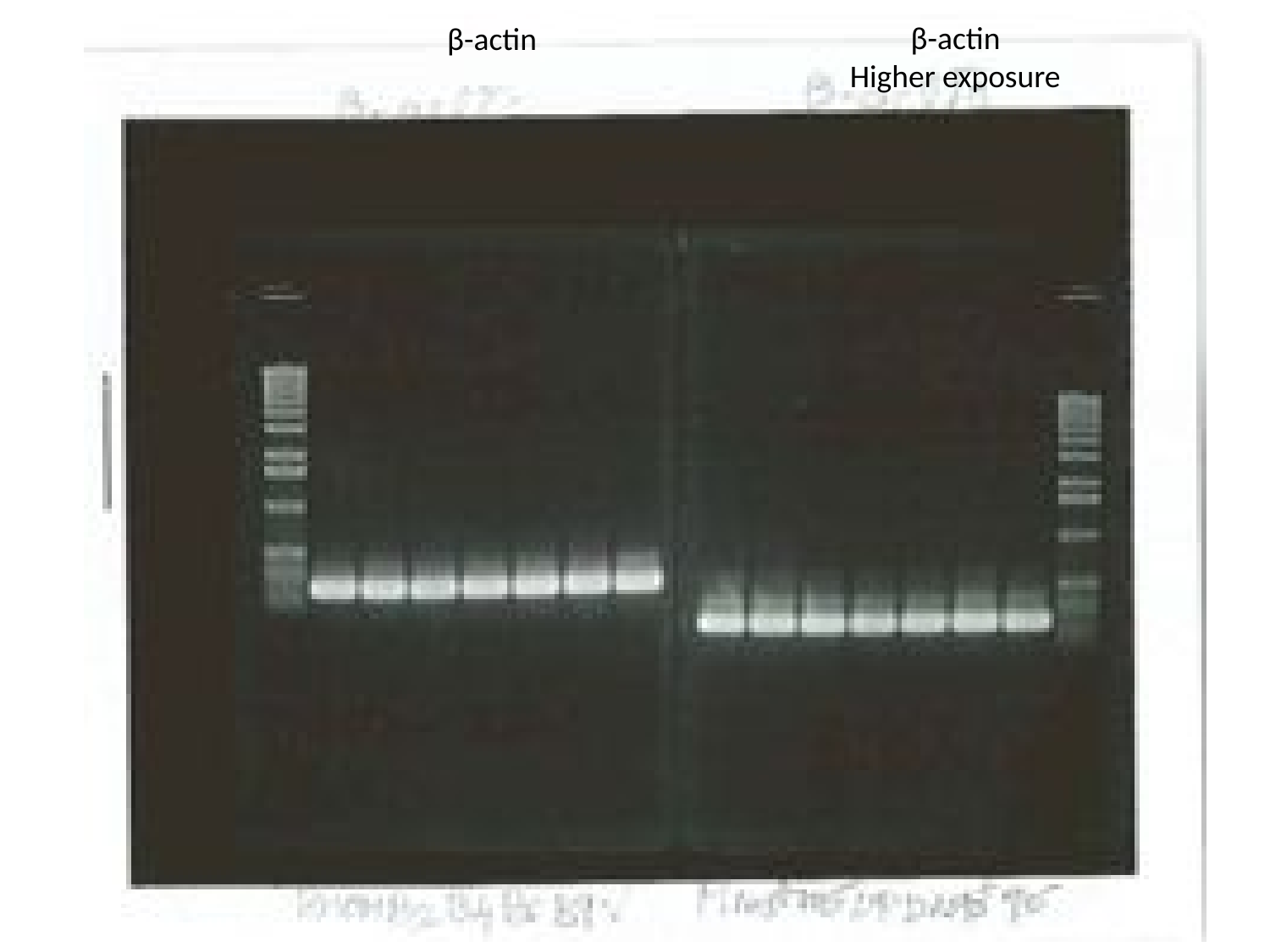

β-actin
Higher exposure
β-actin

## Slide 7
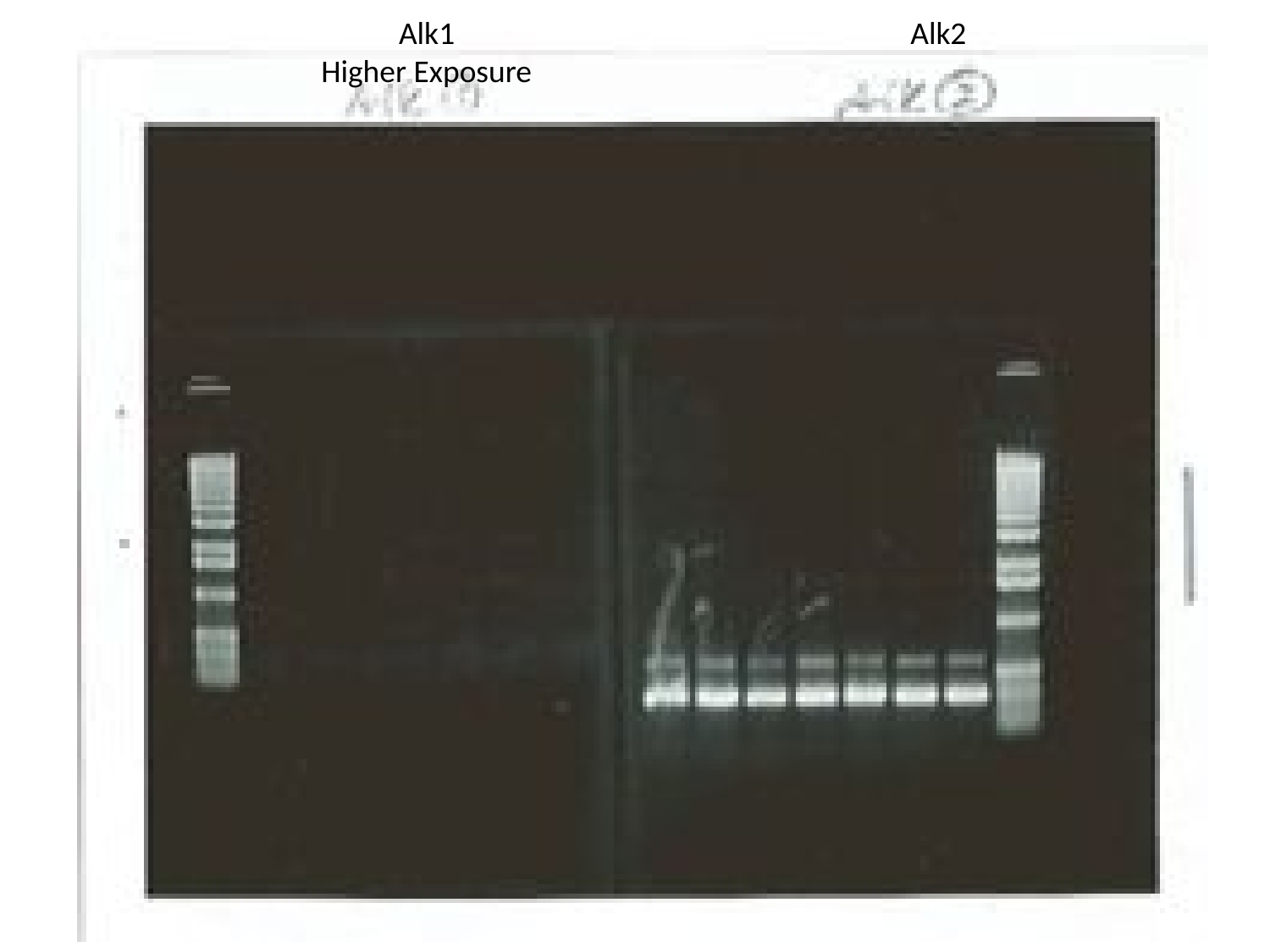

Alk1
Higher Exposure
Alk2
