# Supplementary material for: Correction: BMP-Non-Responsive Sca1+CD73+CD44+ Mouse Bone Marrow Derived Osteoprogenitor Cells Respond to Combination of VEGF and BMP-6 to Display Enhanced Osteoblastic Differentiation and Ectopic Bone Formation
Source: PLoS One. 2019 Jan 31;14(1):e0211782. doi: 10.1371/journal.pone.0211782 (PMC6355026; doi:10.1371/journal.pone.0211782)
Supplement: S4 Data — (PPTX) [file pone.0211782.s005.pptx]

## Slide 1
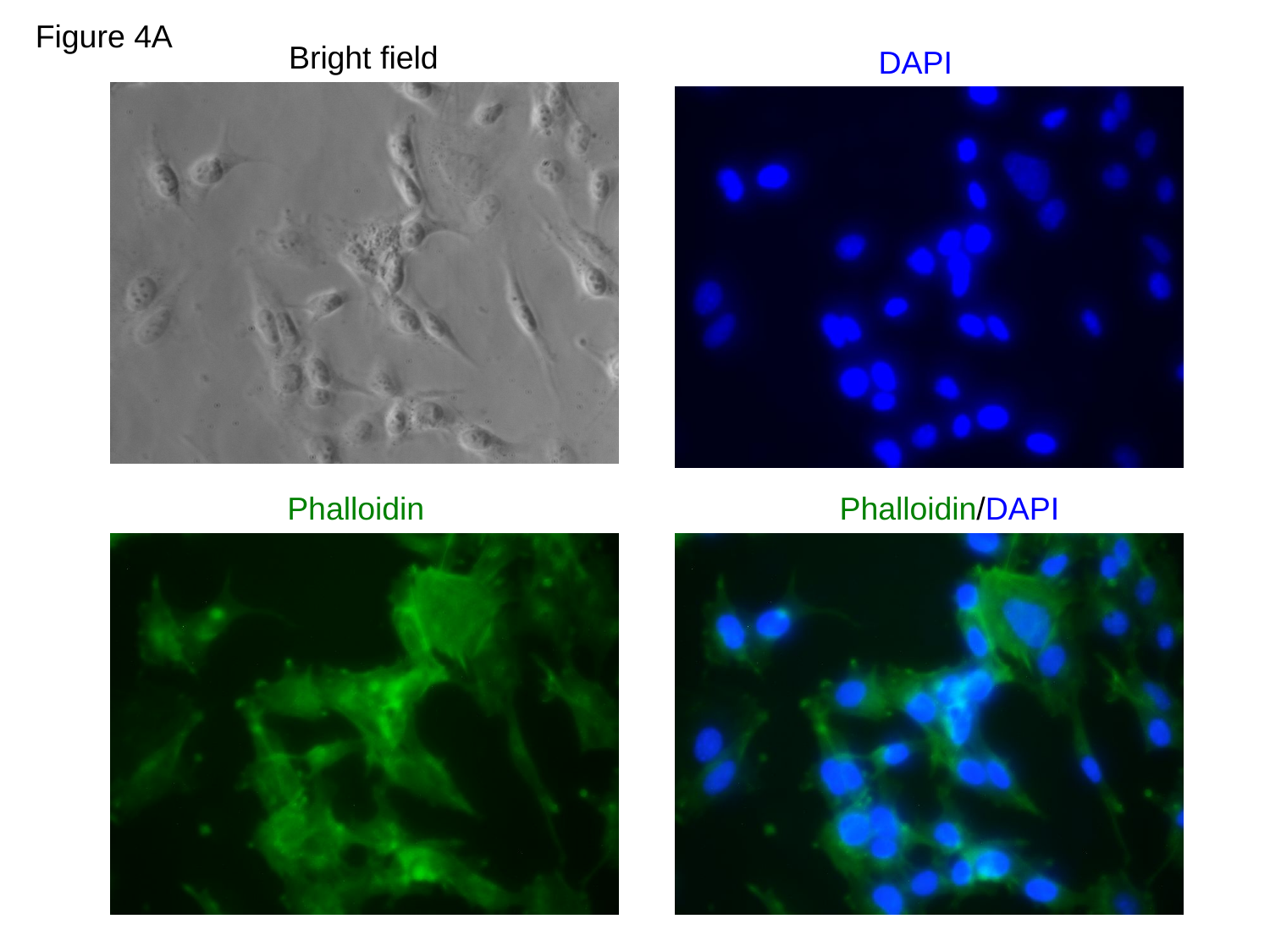

Figure 4A
Bright field
DAPI
Phalloidin
Phalloidin/DAPI

## Slide 2
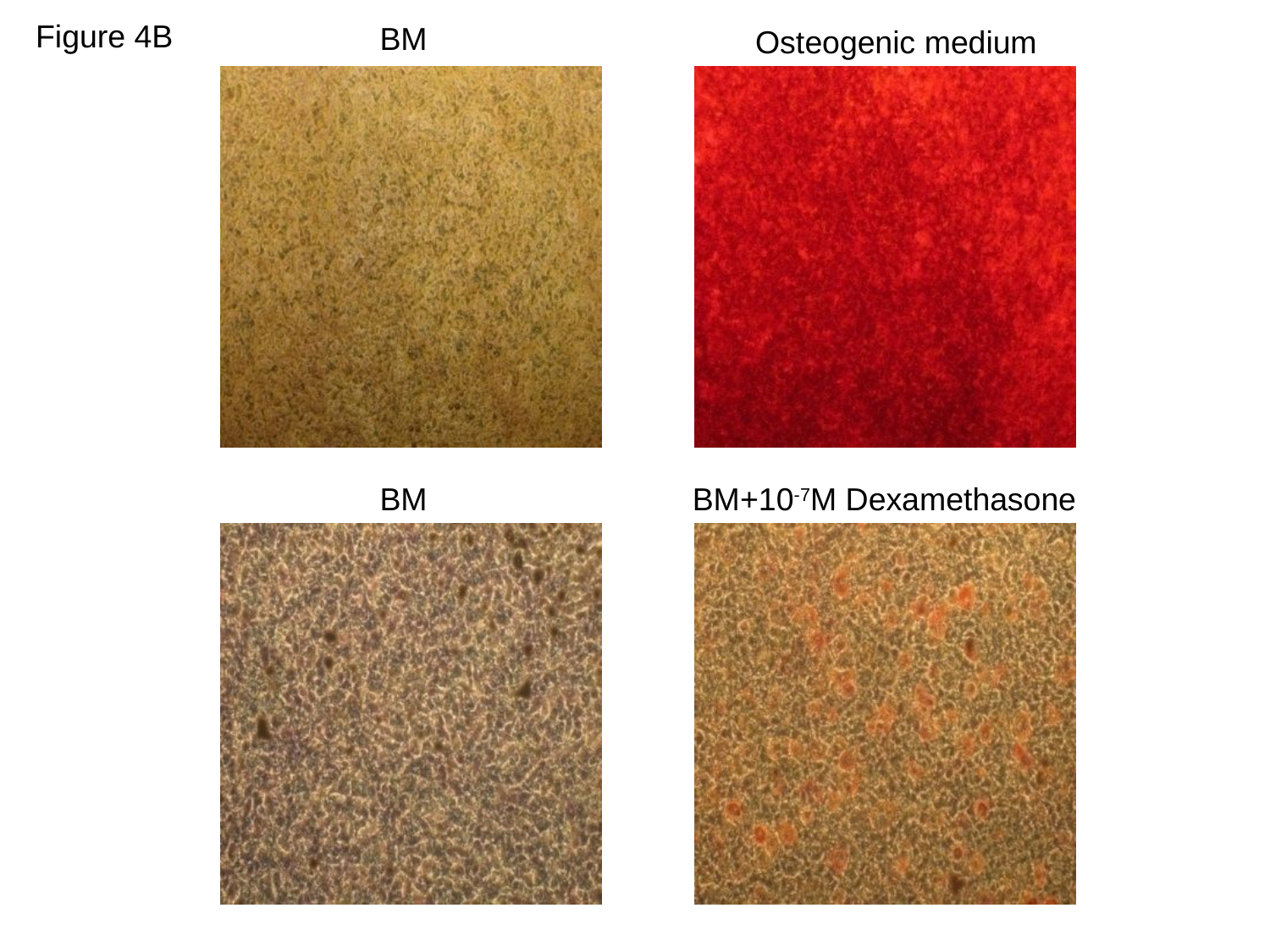

Figure 4B
BM
Osteogenic medium
BM
BM+10-7M Dexamethasone
