# Supplementary material for: Correction: BMP-Non-Responsive Sca1+CD73+CD44+ Mouse Bone Marrow Derived Osteoprogenitor Cells Respond to Combination of VEGF and BMP-6 to Display Enhanced Osteoblastic Differentiation and Ectopic Bone Formation
Source: PLoS One. 2019 Jan 31;14(1):e0211782. doi: 10.1371/journal.pone.0211782 (PMC6355026; doi:10.1371/journal.pone.0211782)
Supplement: S5 Data — (ZIP) [file pone.0211782.s006.zip › Figure5Statistics.docx]

| **ALP/18s** | | | | | |
| --- | --- | --- | --- | --- | --- |
| **ANOVA** | | | | | |
| data | | | | | |
|  | 平方和 | df | 均方 | F | 显著性 |
| 组间 | .237 | 4 | .059 | 28.256 | .001 |
| 组内 | .010 | 5 | .002 |  |  |
| 总数 | .248 | 9 |  |  |  |

| **多重比较** | | | | | | | | |
| --- | --- | --- | --- | --- | --- | --- | --- | --- |
| data  LSD | | | | | | | | |
| (I) group | | (J) group | | 均值差 (I-J) | 标准误 | 显著性 | 95% 置信区间 | |
|  |  |  |  |  |  |  | 下限 | 上限 |
| dimension2 | 1 | dimension3 | 2 | .040277247 | .045819908 | .420 | -.07750658 | .15806107 |
|  |  |  | 3 | .062441552 | .045819908 | .231 | -.05534227 | .18022537 |
|  |  |  | 4 | .072888547 | .045819908 | .173 | -.04489528 | .19067237 |
|  |  |  | 5 | -.336099133^*^ | .045819908 | .001 | -.45388296 | -.21831531 |
|  | 2 | dimension3 | 1 | -.040277247 | .045819908 | .420 | -.15806107 | .07750658 |
|  |  |  | 3 | .022164305 | .045819908 | .649 | -.09561952 | .13994813 |
|  |  |  | 4 | .032611300 | .045819908 | .508 | -.08517252 | .15039512 |
|  |  |  | 5 | -.376376379^*^ | .045819908 | .000 | -.49416020 | -.25859256 |
|  | 3 | dimension3 | 1 | -.062441552 | .045819908 | .231 | -.18022537 | .05534227 |
|  |  |  | 2 | -.022164305 | .045819908 | .649 | -.13994813 | .09561952 |
|  |  |  | 4 | .010446996 | .045819908 | .829 | -.10733683 | .12823082 |
|  |  |  | 5 | -.398540684^*^ | .045819908 | .000 | -.51632451 | -.28075686 |
|  | 4 | dimension3 | 1 | -.072888547 | .045819908 | .173 | -.19067237 | .04489528 |
|  |  |  | 2 | -.032611300 | .045819908 | .508 | -.15039512 | .08517252 |
|  |  |  | 3 | -.010446996 | .045819908 | .829 | -.12823082 | .10733683 |
|  |  |  | 5 | -.408987680^*^ | .045819908 | .000 | -.52677150 | -.29120386 |
|  | 5 | dimension3 | 1 | .336099133^*^ | .045819908 | .001 | .21831531 | .45388296 |
|  |  |  | 2 | .376376379^*^ | .045819908 | .000 | .25859256 | .49416020 |
|  |  |  | 3 | .398540684^*^ | .045819908 | .000 | .28075686 | .51632451 |
|  |  |  | 4 | .408987680^*^ | .045819908 | .000 | .29120386 | .52677150 |
| *. 均值差的显著性水平为 0.05。 | | | | | | | | |

| **Col/18s** | | | | | |
| --- | --- | --- | --- | --- | --- |
| **ANOVA** | | | | | |
| data | | | | | |
|  | 平方和 | df | 均方 | F | 显著性 |
| 组间 | 1.816 | 4 | .454 | 32.768 | .001 |
| 组内 | .069 | 5 | .014 |  |  |
| 总数 | 1.885 | 9 |  |  |  |

| **多重比较** | | | | | | | | |
| --- | --- | --- | --- | --- | --- | --- | --- | --- |
| data  LSD | | | | | | | | |
| (I) group | | (J) group | | 均值差 (I-J) | 标准误 | 显著性 | 95% 置信区间 | |
|  |  |  |  |  |  |  | 下限 | 上限 |
| dimension2 | 1 | dimension3 | 2 | .277594830 | .117710225 | .065 | -.02498893 | .58017860 |
|  |  |  | 3 | .190895928 | .117710225 | .166 | -.11168784 | .49347969 |
|  |  |  | 4 | .476940736^*^ | .117710225 | .010 | .17435697 | .77952450 |
|  |  |  | 5 | -.757713453^*^ | .117710225 | .001 | -1.06029722 | -.45512969 |
|  | 2 | dimension3 | 1 | -.277594830 | .117710225 | .065 | -.58017860 | .02498893 |
|  |  |  | 3 | -.086698902 | .117710225 | .494 | -.38928267 | .21588486 |
|  |  |  | 4 | .199345906 | .117710225 | .151 | -.10323786 | .50192967 |
|  |  |  | 5 | -1.035308283^*^ | .117710225 | .000 | -1.33789205 | -.73272452 |
|  | 3 | dimension3 | 1 | -.190895928 | .117710225 | .166 | -.49347969 | .11168784 |
|  |  |  | 2 | .086698902 | .117710225 | .494 | -.21588486 | .38928267 |
|  |  |  | 4 | .286044808 | .117710225 | .059 | -.01653896 | .58862857 |
|  |  |  | 5 | -.948609381^*^ | .117710225 | .000 | -1.25119315 | -.64602562 |
|  | 4 | dimension3 | 1 | -.476940736^*^ | .117710225 | .010 | -.77952450 | -.17435697 |
|  |  |  | 2 | -.199345906 | .117710225 | .151 | -.50192967 | .10323786 |
|  |  |  | 3 | -.286044808 | .117710225 | .059 | -.58862857 | .01653896 |
|  |  |  | 5 | -1.234654189^*^ | .117710225 | .000 | -1.53723795 | -.93207042 |
|  | 5 | dimension3 | 1 | .757713453^*^ | .117710225 | .001 | .45512969 | 1.06029722 |
|  |  |  | 2 | 1.035308283^*^ | .117710225 | .000 | .73272452 | 1.33789205 |
|  |  |  | 3 | .948609381^*^ | .117710225 | .000 | .64602562 | 1.25119315 |
|  |  |  | 4 | 1.234654189^*^ | .117710225 | .000 | .93207042 | 1.53723795 |
| *. 均值差的显著性水平为 0.05。 | | | | | | | | |

| **Runx2/18s** | | | | | |
| --- | --- | --- | --- | --- | --- |
| **GROUPS**   \| 1BM  2 OM \| 3 BMP6 \| 4 VEGF \| 5 B:V \| \| --- \| --- \| --- \| --- \| | | | | | |
| **ANOVA** | | | | | |
| data | | | | | |
|  | 平方和 | df | 均方 | F | 显著性 |
| 组间 | .317 | 4 | .079 | 2.283 | .195 |
| 组内 | .173 | 5 | .035 |  |  |
| 总数 | .490 | 9 |  |  |  |

| **多重比较** | | | | | | | | |
| --- | --- | --- | --- | --- | --- | --- | --- | --- |
| data  LSD | | | | | | | | |
| (I) group | | (J) group | | 均值差 (I-J) | 标准误 | 显著性 | 95% 置信区间 | |
|  |  |  |  |  |  |  | 下限 | 上限 |
| dimension2 | 1 | dimension3 | 2 | .411951023 | .186265787 | .078 | -.06686043 | .89076247 |
|  |  |  | 3 | .093130154 | .186265787 | .638 | -.38568130 | .57194160 |
|  |  |  | 4 | .435778651 | .186265787 | .066 | -.04303280 | .91459010 |
|  |  |  | 5 | .115761128 | .186265787 | .562 | -.36305032 | .59457258 |
|  | 2 | dimension3 | 1 | -.411951023 | .186265787 | .078 | -.89076247 | .06686043 |
|  |  |  | 3 | -.318820869 | .186265787 | .148 | -.79763232 | .15999058 |
|  |  |  | 4 | .023827628 | .186265787 | .903 | -.45498382 | .50263908 |
|  |  |  | 5 | -.296189895 | .186265787 | .173 | -.77500134 | .18262155 |
|  | 3 | dimension3 | 1 | -.093130154 | .186265787 | .638 | -.57194160 | .38568130 |
|  |  |  | 2 | .318820869 | .186265787 | .148 | -.15999058 | .79763232 |
|  |  |  | 4 | .342648497 | .186265787 | .125 | -.13616295 | .82145995 |
|  |  |  | 5 | .022630974 | .186265787 | .908 | -.45618048 | .50144242 |
|  | 4 | dimension3 | 1 | -.435778651 | .186265787 | .066 | -.91459010 | .04303280 |
|  |  |  | 2 | -.023827628 | .186265787 | .903 | -.50263908 | .45498382 |
|  |  |  | 3 | -.342648497 | .186265787 | .125 | -.82145995 | .13616295 |
|  |  |  | 5 | -.320017523 | .186265787 | .146 | -.79882897 | .15879393 |
|  | 5 | dimension3 | 1 | -.115761128 | .186265787 | .562 | -.59457258 | .36305032 |
|  |  |  | 2 | .296189895 | .186265787 | .173 | -.18262155 | .77500134 |
|  |  |  | 3 | -.022630974 | .186265787 | .908 | -.50144242 | .45618048 |
|  |  |  | 4 | .320017523 | .186265787 | .146 | -.15879393 | .79882897 |

| **Osx/18s** | | | | | |
| --- | --- | --- | --- | --- | --- |
| **ANOVA** | | | | | |
| data | | | | | |
|  | 平方和 | df | 均方 | F | 显著性 |
| 组间 | 1.130 | 4 | .282 | 18.781 | .003 |
| 组内 | .075 | 5 | .015 |  |  |
| 总数 | 1.205 | 9 |  |  |  |

| **多重比较** | | | | | | | | |
| --- | --- | --- | --- | --- | --- | --- | --- | --- |
| data  LSD | | | | | | | | |
| (I) group | | (J) group | | 均值差 (I-J) | 标准误 | 显著性 | 95% 置信区间 | |
|  |  |  |  |  |  |  | 下限 | 上限 |
| dimension2 | 1 | dimension3 | 2 | -.287652380 | .122620070 | .066 | -.60285730 | .02755255 |
|  |  |  | 3 | -.067637454 | .122620070 | .605 | -.38284238 | .24756747 |
|  |  |  | 4 | -.065573123 | .122620070 | .616 | -.38077805 | .24963180 |
|  |  |  | 5 | -.909447483^*^ | .122620070 | .001 | -1.22465241 | -.59424256 |
|  | 2 | dimension3 | 1 | .287652380 | .122620070 | .066 | -.02755255 | .60285730 |
|  |  |  | 3 | .220014926 | .122620070 | .133 | -.09519000 | .53521985 |
|  |  |  | 4 | .222079257 | .122620070 | .130 | -.09312567 | .53728418 |
|  |  |  | 5 | -.621795104^*^ | .122620070 | .004 | -.93700003 | -.30659018 |
|  | 3 | dimension3 | 1 | .067637454 | .122620070 | .605 | -.24756747 | .38284238 |
|  |  |  | 2 | -.220014926 | .122620070 | .133 | -.53521985 | .09519000 |
|  |  |  | 4 | .002064331 | .122620070 | .987 | -.31314059 | .31726926 |
|  |  |  | 5 | -.841810029^*^ | .122620070 | .001 | -1.15701495 | -.52660510 |
|  | 4 | dimension3 | 1 | .065573123 | .122620070 | .616 | -.24963180 | .38077805 |
|  |  |  | 2 | -.222079257 | .122620070 | .130 | -.53728418 | .09312567 |
|  |  |  | 3 | -.002064331 | .122620070 | .987 | -.31726926 | .31314059 |
|  |  |  | 5 | -.843874361^*^ | .122620070 | .001 | -1.15907929 | -.52866944 |
|  | 5 | dimension3 | 1 | .909447483^*^ | .122620070 | .001 | .59424256 | 1.22465241 |
|  |  |  | 2 | .621795104^*^ | .122620070 | .004 | .30659018 | .93700003 |
|  |  |  | 3 | .841810029^*^ | .122620070 | .001 | .52660510 | 1.15701495 |
|  |  |  | 4 | .843874361^*^ | .122620070 | .001 | .52866944 | 1.15907929 |
| *. 均值差的显著性水平为 0.05。 | | | | | | | | |
