# Supplementary material for: Correction: BMP-Non-Responsive Sca1+CD73+CD44+ Mouse Bone Marrow Derived Osteoprogenitor Cells Respond to Combination of VEGF and BMP-6 to Display Enhanced Osteoblastic Differentiation and Ectopic Bone Formation
Source: PLoS One. 2019 Jan 31;14(1):e0211782. doi: 10.1371/journal.pone.0211782 (PMC6355026; doi:10.1371/journal.pone.0211782)
Supplement: S6 Data — (ZIP) [file pone.0211782.s007.zip › Figure6Statistics.docx]

| **2 weeks implant** | | | | | |
| --- | --- | --- | --- | --- | --- |
| **Group**   \| 1 OM \| 2 BMP6 \| 3 VEGF \| 4 B:V \| \| --- \| --- \| --- \| --- \| | | | | | |
| **ANOVA** | | | | | |
| data | | | | | |
|  | 平方和 | df | 均方 | F | 显著性 |
| 组间 | .057 | 3 | .019 | .589 | .630 |
| 组内 | .651 | 20 | .033 |  |  |
| 总数 | .709 | 23 |  |  |  |

| **多重比较** | | | | | | | | |
| --- | --- | --- | --- | --- | --- | --- | --- | --- |
| data  LSD | | | | | | | | |
| (I) group | | (J) group | | 均值差 (I-J) | 标准误 | 显著性 | 95% 置信区间 | |
|  |  |  |  |  |  |  | 下限 | 上限 |
| dimension2 | 1 | dimension3 | 2 | .063724731 | .110489487 | .571 | -.16675230 | .29420176 |
|  |  |  | 3 | .097461890 | .110489487 | .388 | -.13301514 | .32793892 |
|  |  |  | 4 | -.033730932 | .090214288 | .712 | -.22191464 | .15445278 |
|  | 2 | dimension3 | 1 | -.063724731 | .110489487 | .571 | -.29420176 | .16675230 |
|  |  |  | 3 | .033737160 | .127582270 | .794 | -.23239479 | .29986911 |
|  |  |  | 4 | -.097455662 | .110489487 | .388 | -.32793269 | .13302137 |
|  | 3 | dimension3 | 1 | -.097461890 | .110489487 | .388 | -.32793892 | .13301514 |
|  |  |  | 2 | -.033737160 | .127582270 | .794 | -.29986911 | .23239479 |
|  |  |  | 4 | -.131192822 | .110489487 | .249 | -.36166985 | .09928421 |
|  | 4 | dimension3 | 1 | .033730932 | .090214288 | .712 | -.15445278 | .22191464 |
|  |  |  | 2 | .097455662 | .110489487 | .388 | -.13302137 | .32793269 |
|  |  |  | 3 | .131192822 | .110489487 | .249 | -.09928421 | .36166985 |

**4weeks**

| **ANOVA** | | | | | |
| --- | --- | --- | --- | --- | --- |
| data | | | | | |
|  | 平方和 | df | 均方 | F | 显著性 |
| 组间 | .351 | 3 | .117 | 2.828 | .057 |
| 组内 | 1.159 | 28 | .041 |  |  |
| 总数 | 1.510 | 31 |  |  |  |

| **多重比较** | | | | | | | | |
| --- | --- | --- | --- | --- | --- | --- | --- | --- |
| data  LSD | | | | | | | | |
| (I) group | | (J) group | | 均值差 (I-J) | 标准误 | 显著性 | 95% 置信区间 | |
|  |  |  |  |  |  |  | 下限 | 上限 |
| dimension2 | 1 | dimension3 | 2 | -.082629149 | .101728216 | .423 | -.29100995 | .12575165 |
|  |  |  | 3 | -.021731144 | .101728216 | .832 | -.23011195 | .18664966 |
|  |  |  | 4 | -.266402490^*^ | .101728216 | .014 | -.47478329 | -.05802169 |
|  | 2 | dimension3 | 1 | .082629149 | .101728216 | .423 | -.12575165 | .29100995 |
|  |  |  | 3 | .060898005 | .101728216 | .554 | -.14748280 | .26927881 |
|  |  |  | 4 | -.183773341 | .101728216 | .082 | -.39215414 | .02460746 |
|  | 3 | dimension3 | 1 | .021731144 | .101728216 | .832 | -.18664966 | .23011195 |
|  |  |  | 2 | -.060898005 | .101728216 | .554 | -.26927881 | .14748280 |
|  |  |  | 4 | -.244671346^*^ | .101728216 | .023 | -.45305215 | -.03629054 |
|  | 4 | dimension3 | 1 | .266402490^*^ | .101728216 | .014 | .05802169 | .47478329 |
|  |  |  | 2 | .183773341 | .101728216 | .082 | -.02460746 | .39215414 |
|  |  |  | 3 | .244671346^*^ | .101728216 | .023 | .03629054 | .45305215 |
| *. 均值差的显著性水平为 0.05。 | | | | | | | | |

| **2weeks OPG/RANKL** | | | | | |
| --- | --- | --- | --- | --- | --- |
| **ANOVA** | | | | | |
| data | | | | | |
|  | 平方和 | df | 均方 | F | P |
| 组间 | 1973.353 | 3 | 657.784 | 3.261 | .142 |
| 组内 | 806.771 | 4 | 201.693 |  |  |
| 总数 | 2780.124 | 7 |  |  |  |

| **多重比较** | | | | | | | | | |
| --- | --- | --- | --- | --- | --- | --- | --- | --- | --- |
| 因变量:data | | | | | | | | | |
|  | (I) group | | (J) group | | 均值差 (I-J) | 标准误 | **Sig P** | 95% 置信区间 | |
|  |  |  |  |  |  |  |  | 下限 | 上限 |
| **LSD** | dimension2 | 1 | dimension3 | 2 | -38.2482556 | 14.2018560 | **.054** | -77.678929 | 1.182418 |
|  |  |  |  | 3 | -35.1257374 | 14.2018560 | **.069** | -74.556411 | 4.304936 |
|  |  |  |  | 4 | -13.8428494 | 14.2018560 | **.385** | -53.273523 | 25.587824 |
|  |  | 2 | dimension3 | 1 | 38.2482556 | 14.2018560 | **.054** | -1.182418 | 77.678929 |
|  |  |  |  | 3 | 3.1225182 | 14.2018560 | **.837** | -36.308155 | 42.553192 |
|  |  |  |  | 4 | 24.4054061 | 14.2018560 | **.161** | -15.025267 | 63.836080 |
|  |  | 3 | dimension3 | 1 | 35.1257374 | 14.2018560 | **.069** | -4.304936 | 74.556411 |
|  |  |  |  | 2 | -3.1225182 | 14.2018560 | **.837** | -42.553192 | 36.308155 |
|  |  |  |  | 4 | 21.2828880 | 14.2018560 | **.208** | -18.147786 | 60.713562 |
|  |  | 4 | dimension3 | 1 | 13.8428494 | 14.2018560 | **.385** | -25.587824 | 53.273523 |
|  |  |  |  | 2 | -24.4054061 | 14.2018560 | **.161** | -63.836080 | 15.025267 |
|  |  |  |  | 3 | -21.2828880 | 14.2018560 | **.208** | -60.713562 | 18.147786 |

| **4weeks OPG/RANKL** | | | | | |
| --- | --- | --- | --- | --- | --- |
|  | | | | | |
| **ANOVA** | | | | | |
| data | | | | | |
|  | 平方和 | df | 均方 | F | p |
| 组间 | 105.275 | 3 | 35.092 | 10.850 | .022 |
| 组内 | 12.937 | 4 | 3.234 |  |  |
| 总数 | 118.212 | 7 |  |  |  |

| **多重比较** | | | | | | | | | |
| --- | --- | --- | --- | --- | --- | --- | --- | --- | --- |
| 因变量:data | | | | | | | | | |
|  | (I) group | | (J) group | | 均值差 (I-J) | 标准误 | Sig P | 95% 置信区间 | |
|  |  |  |  |  |  |  |  | 下限 | 上限 |
| **LSD** | dimension2 | 1 | dimension3 | 2 | .5957512 | 1.7984182 | **.757** | -4.397458 | 5.588961 |
|  |  |  |  | 3 | -.3913853 | 1.7984182 | **.838** | -5.384595 | 4.601824 |
|  |  |  |  | 4 | -8.2700066^*^ | 1.7984182 | **.010** | -13.263216 | -3.276797 |
|  |  | 2 | dimension3 | 1 | -.5957512 | 1.7984182 | **.757** | -5.588961 | 4.397458 |
|  |  |  |  | 3 | -.9871365 | 1.7984182 | **.612** | -5.980346 | 4.006073 |
|  |  |  |  | 4 | -8.8657578^*^ | 1.7984182 | **.008** | -13.858967 | -3.872548 |
|  |  | 3 | dimension3 | 1 | .3913853 | 1.7984182 | **.838** | -4.601824 | 5.384595 |
|  |  |  |  | 2 | .9871365 | 1.7984182 | **.612** | -4.006073 | 5.980346 |
|  |  |  |  | 4 | -7.8786213^*^ | 1.7984182 | **.012** | -12.871831 | -2.885412 |
|  |  | 4 | dimension3 | 1 | 8.2700066^*^ | 1.7984182 | **.010** | 3.276797 | 13.263216 |
|  |  |  |  | 2 | 8.8657578^*^ | 1.7984182 | **.008** | 3.872548 | 13.858967 |
|  |  |  |  | 3 | 7.8786213^*^ | 1.7984182 | **.012** | 2.885412 | 12.871831 |
| *. 均值差的显著性水平为 0.05。 | | | | | | | | | |
