# Supplementary material for: Correction: BMP-Non-Responsive Sca1+CD73+CD44+ Mouse Bone Marrow Derived Osteoprogenitor Cells Respond to Combination of VEGF and BMP-6 to Display Enhanced Osteoblastic Differentiation and Ectopic Bone Formation
Source: PLoS One. 2019 Jan 31;14(1):e0211782. doi: 10.1371/journal.pone.0211782 (PMC6355026; doi:10.1371/journal.pone.0211782)
Supplement: S8 Data — (ZIP) [file pone.0211782.s009.zip › Figure7Statistics.docx]

| **2 weeks** | | | | | |
| --- | --- | --- | --- | --- | --- |
| **CADM1/18s** | | | | | |
| **ANOVA** | | | | | |
| data | | | | | |
|  | 平方和 | df | 均方 | F | 显著性 |
| 组间 | .034 | 3 | .011 | 4.875 | .080 |
| 组内 | .009 | 4 | .002 |  |  |
| 总数 | .044 | 7 |  |  |  |

| **多重比较** | | | | | | | | |
| --- | --- | --- | --- | --- | --- | --- | --- | --- |
| data  LSD | | | | | | | | |
| (I) group | | (J) group | | 均值差 (I-J) | 标准误 | 显著性 | 95% 置信区间 | |
|  |  |  |  |  |  |  | 下限 | 上限 |
| dimension2 | 1 | dimension3 | 2 | -.166393560^*^ | .048351335 | .026 | -.30063839 | -.03214873 |
|  |  |  | 3 | -.074408702 | .048351335 | .199 | -.20865353 | .05983612 |
|  |  |  | 4 | -.014947459 | .048351335 | .773 | -.14919229 | .11929737 |
|  | 2 | dimension3 | 1 | .166393560^*^ | .048351335 | .026 | .03214873 | .30063839 |
|  |  |  | 3 | .091984858 | .048351335 | .130 | -.04225997 | .22622969 |
|  |  |  | 4 | .151446102^*^ | .048351335 | .035 | .01720128 | .28569093 |
|  | 3 | dimension3 | 1 | .074408702 | .048351335 | .199 | -.05983612 | .20865353 |
|  |  |  | 2 | -.091984858 | .048351335 | .130 | -.22622969 | .04225997 |
|  |  |  | 4 | .059461244 | .048351335 | .286 | -.07478358 | .19370607 |
|  | 4 | dimension3 | 1 | .014947459 | .048351335 | .773 | -.11929737 | .14919229 |
|  |  |  | 2 | -.151446102^*^ | .048351335 | .035 | -.28569093 | -.01720128 |
|  |  |  | 3 | -.059461244 | .048351335 | .286 | -.19370607 | .07478358 |
| *. 均值差的显著性水平为 0.05。 | | | | | | | | |

| **4 weeks** |
| --- |
| **CADM1/18s** |

| **ANOVA** | | | | | |
| --- | --- | --- | --- | --- | --- |
| data | | | | | |
|  | 平方和 | df | 均方 | F | 显著性 |
| 组间 | .142 | 3 | .047 | 56.342 | .000 |
| 组内 | .007 | 8 | .001 |  |  |
| 总数 | .149 | 11 |  |  |  |

| **多重比较** | | | | | | | | |
| --- | --- | --- | --- | --- | --- | --- | --- | --- |
| data  LSD | | | | | | | | |
| (I) group | | (J) group | | 均值差 (I-J) | 标准误 | 显著性 | 95% 置信区间 | |
|  |  |  |  |  |  |  | 下限 | 上限 |
| dimension2 | 1 | dimension3 | 2 | .052735041 | .023650381 | .056 | -.00180284 | .10727292 |
|  |  |  | 3 | -.235802986^*^ | .023650381 | .000 | -.29034086 | -.18126511 |
|  |  |  | 4 | -.069876926^*^ | .023650381 | .018 | -.12441480 | -.01533905 |
|  | 2 | dimension3 | 1 | -.052735041 | .023650381 | .056 | -.10727292 | .00180284 |
|  |  |  | 3 | -.288538027^*^ | .023650381 | .000 | -.34307590 | -.23400015 |
|  |  |  | 4 | -.122611966^*^ | .023650381 | .001 | -.17714984 | -.06807409 |
|  | 3 | dimension3 | 1 | .235802986^*^ | .023650381 | .000 | .18126511 | .29034086 |
|  |  |  | 2 | .288538027^*^ | .023650381 | .000 | .23400015 | .34307590 |
|  |  |  | 4 | .165926060^*^ | .023650381 | .000 | .11138818 | .22046394 |
|  | 4 | dimension3 | 1 | .069876926^*^ | .023650381 | .018 | .01533905 | .12441480 |
|  |  |  | 2 | .122611966^*^ | .023650381 | .001 | .06807409 | .17714984 |
|  |  |  | 3 | -.165926060^*^ | .023650381 | .000 | -.22046394 | -.11138818 |
| *. 均值差的显著性水平为 0.05。 | | | | | | | | |

1 OM

2 BMP6

3 VEGF

4 B6+V
